# Impact of *Fomes fomentarius* growth on the mechanical properties of material extrusion additively manufactured PLA and PLA/Hemp biopolymers

**DOI:** 10.1186/s40694-025-00205-9

**Published:** 2025-11-08

**Authors:** Narges Panjalipoursangari, Yating Ou, Bertram Schmidt, Wolfgang H. Müller, Christina Völlmecke

**Affiliations:** 1 Continuum Mechanics and Materials Theory Group, Technical University of Berlin, Einsteinufer 5, 10587 Berlin, Germany; 2 Stability and Failure of Functionally Optimized Structures Group, Technical University of Berlin, Einsteinufer 5, 10587 Berlin, Germany; 3 Applied and Molecular Microbiology Group, Technical University of Berlin, Gustav-Meyer-Allee 25D, 13355 Berlin, Germany

**Keywords:** Mycelium-based composites, Material extrusion additive manufacturing (MEX AM), Mechanical characterization, Tensile behavior, Sustainability

## Abstract

Fungal-based biomaterials are emerging as sustainable alternatives to synthetic polymers, offering biodegradability and low environmental impact. However, the interaction between mycelium and 3D-printed biopolymers, particularly regarding mechanical performance, remains underexplored. This research investigates the tensile behavior of biopolymer specimens produced by Material Extrusion Additive Manufacturing (MEX AM), focusing on the effects of *Fomes fomentarius* mycelium colonization. The study examines how pre- and post-processing steps, as well as different 3D-printing infill patterns, influence mycelial growth and its mechanical impact. Both pure PLA and PLA_Hemp biopolymers were studied to assess the role of natural particles in fungal interaction and structural performance. The results indicate that mycelial colonization has a minor impact on the mechanical properties of PLA, while PLA_Hemp shows more pronounced, time-dependent effects. Environmental conditions such as humidity and incubation also affect mechanical performance, whereas certain pretreatments, like autoclaving, can significantly weaken the material. Overall, this work provides insight into the integration of mycelium within 3D-printing biopolymers, demonstrating the feasibility of hybrid biocomposites and highlighting both opportunities and challenges, thereby paving the way for more sustainable materials design and construction practices.

## Introduction

Fungal-derived biomaterials are increasingly recognized as promising alternatives to petroleum-based products in various sectors such as packaging, textiles, and construction materials [1, 2]. This shift is driven by the unique enzymatic capabilities of fungi, particularly in degrading lignocellulosic substrates from agricultural and forestry residues, thereby addressing critical challenges in waste management [3, 4]. These biomaterials not only offer renewable alternatives but also align with the United Nations Sustainable Development Goals, particularly Sustainable Cities and Communities (SDG 11), Responsible Consumption and Production (SDG 12), and Climate Action (SDG 13) [3, 4].

Research efforts have predominantly centered on fungal species from genera like *Ganoderma*, *Pleurotus* and *Trametes*, known for their ability to produce a variety of pure mycelium or fungal-based composite materials. Applications span a wide range, encompassing packaging, thermal and acoustic insulation, construction materials, and ongoing investigations into potential alternatives for leather [1, 2]. Detailed reviews provide comprehensive insights into their production methods and material properties, crucial for advancing their industrial applications [1, 2].

One notable example in fungal biomaterial innovation is *Fomes fomentarius*, a terrestrial white-rot polypore commonly known as the tinder fungus. This species occurs naturally on various deciduous trees as a parasite and saprobiont, but in technical solid-state cultivations it can also grow on many other lignocellulosic substrates such as hemp shives, rapeseed straw, and poplar sawdust. The resulting robust composite materials show competitive potential compared to traditional materials like concrete, particularly with regard to environmental impact assessments [3, 4]. Lifecycle assessments have highlighted significant environmental advantages of composite bricks made from *Fomes fomentarius*-derived materials, demonstrating reduced impacts on climate change, smog, and water scarcity when compared to conventional building materials [5]. These findings underscore the potential of fungal-based composites to mitigate environmental impacts in construction practices and foster sustainable building methods.

Although considerable progress has been made in developing fungal biomaterials, critical knowledge gaps remain. In particular, the interaction between 3D-printed structures and fungal colonization remains underexplored, especially regarding the effect of infill pattern and colonization time on the tensile properties of PLA and PLA_Hemp composites. Furthermore, specific mechanisms such as interface bonding and anisotropy induced by infill orientation have not yet been fully elucidated. Addressing these gaps is crucial for designing biohybrid composites that effectively integrate mechanical robustness with the functional advantages of living mycelium.

Recent developments indicate that mycelium from *Fomes fomentarius* can be effectively utilized in extrusion-based 3D-printing, producing composite materials with compressive strength comparable to Expanded PolyStyrene (EPS) [6]. Since EPS is primarily used as an insulating material rather than for load bearing structural components, current mycelium-based composites should likewise be regarded as alternatives for nonstructural applications. Related work on the mechanical characterization of non-printed *Fomes fomentarius* mycelium-based composites has also been reported [7]. Recent studies have shown that 3D-printed gyroid scaffolds made of wood-PLA can be colonized by mycelium, yielding composites that combine thermal insulation with enhanced mechanical performance [8]. In these studies, the scaffolds were placed directly into malt extract agar (MEA), overgrown by *Ganoderma lucidum*, and subsequently characterized with respect to mechanical and thermal properties. These findings highlight the potential of mycelium-based composites for structural applications. However, the fundamental interaction between biopolymers and fungal growth-investigated independently of complex scaffold architectures remains insufficiently understood. In the present work, we address this gap by embedding standardized PLA and PLA_Hemp specimens into pre-grown *Fomes fomentarius* substrates in order to systematically investigate the direct interplay between additively manufactured polymers and living mycelial networks. Our approach follows an integration-into-grown-mycelium-composite paradigm: 3D-printed tensile specimens (PLA and PLA_Hemp) are introduced post-printing into a densely colonized hemp-fines substrate inoculated with *Fomes fomentarius*. To extend the applicability of mycelium-based composites beyond insulation toward mechanically more demanding components, reinforcement strategies such as the targeted integration of biopolymers represent a promising pathway. This approach opens perspectives not only for sustainable insulation solutions but also for the development of bio-based composites with improved structural performance.

Enhanced performance characteristics have so far been demonstrated specifically in heat-pressed mycelium composites composed of *Fomes fomentarius* and hemp shives. In this form, the materials show potential as a sustainable alternative to conventional wood-based particle boards [7].

By contrast, when evaluating the thermal insulation potential of these composites, comparisons are more appropriately drawn with fossil-based insulation materials. Although further improvement in compressive strength is necessary to meet construction standards for external insulation, heat-pressed composites already exhibit thermal conductivity within the range of natural insulation materials, supporting their relevance as eco-friendly alternatives [9].

The present study investigates the fundamental interactions between mycelium and biopolymers in hybrid composites that integrate mycelial substrates with additively manufactured structures, such as 3D-printed lattices. Its primary objective is to establish a foundational understanding of mycelial behavior within different biopolymer matrices, providing a basis for the future development of biohybrid composites. To this end, the research addresses two key questions. First, it examines the effect of different 3D-print patterns (0°, 45°, and grid) on the growth and integration of *Fomes fomentarius* mycelium in PLA and PLA_Hemp matrices. Second, it quantifies how the duration of mycelial colonization affects mechanical properties, focusing on Young’s Modulus and Ultimate Tensile Strength (UTS). By tackling these questions, the study positions itself at the intersection of bio-based material development and additive manufacturing, highlighting current knowledge gaps and motivating the experimental design. Overall, this introduction situates the study within the broader research context, identifies critical gaps regarding mycelium 3D-print interactions, and justifies the focus on mechanical characterization of PLA and PLA_Hemp specimens under controlled colonization conditions. The findings are intended to inform the design of sustainable biohybrid composites that combine structural reliability with functional fungal integration.

## Materials and methods

The experimental methodology involved Material Extrusion Additive Manufacturing (MEX AM) and subsequent tensile testing of the fabricated specimens. Material selection included a specific filament type and the incorporation of *Fomes fomentarius* mycelium, chosen for their relevance to sustainable manufacturing. The modeling process was conducted using computer-aided design (CAD) with parameters adapted for MEX AM. Specimen fabrication steps are described in detail, including post-processing procedures to enable mycelium colonization. The tensile testing setup is specified with attention to force application rates and specimen geometry to ensure reproducibility and accuracy of mechanical characterization.

### Raw filament materials

Polylactic acid (PLA) and a PLA-based composite reinforced with hemp particles (PLA_Hemp) were selected as base materials due to their printability and compatibility with MEX AM [10]. To assess the influence of material composition, specimens were fabricated from both pure PLA and PLA_Hemp using MEX AM. All printed polymer structures (PLA and PLA_Hemp) were subsequently combined with *Fomes fomentarius* mycelium to form hybrid biocomposites. Accordingly, three material categories were considered in this study: (i) MEX-fabricated biopolymer scaffolds, (ii) biologically grown mycelium-based biocomposites, and (iii) hybrid biocomposites integrating printed scaffolds with mycelium. Details of the printing parameters and processing steps are provided in the following subsections. The filaments used in this study included TruePLA Purple Transparent from (FilaFarm [11]), Prusament PLA Galaxy Silver from (Prusa Polymers[12]), and PLA_Hemp Filament from (Canapuglia[13]). Table 1 summarizes the properties of the filaments used. The reported printing properties correspond to the recommended parameters by the manufacturers intended to ensure optimal printability [11–13].

**Table 1:** Material properties of the filaments

	TruePLA purple transparent
Measurement Series	PLA 1, PLA 1_2W, PLA 1_4W
*Material Composition*	100% Polylactic Acid
Nozzle Temperature	195-220°C
*Build Plate Temperature*	0-60°C
Tensile Strength	55 MPa
*Young’s Modulus*	3200 MPa
Diameter	1.75±0.03 mm
*Prusament PLA Galaxy Silver*	
Measurement Series	PLA 2, PLA 2_2W, PLA 2_4W
*Material Composition*	100% Polylactic Acid
Nozzle Temperature	200-220°C
*Build Plate Temperature*	50-60°C
Tensile Strength	51 MPa
*Young’s Modulus*	2300 MPa
Diameter	1.75±0.03 mm
*PLA_Hemp Filament*	
Measurement Series	PLA_Hemp, PLA_Hemp_2W, PLA_Hemp_4W
*Material Composition*	80% Polylactic Acid 20% Hemp Particles
Nozzle Temperature	165-190°C
*Build Plate Temperature*	50°C
Diameter	1.75±0.01 mm

The tensile strength and Young’s modulus values provided by the manufacturers, as listed in Table 1, serve only as approximate guidelines since they stem from injection-moulded specimens. The actual mechanical properties of a 3D-printed specimen, including Young’s modulus and ultimate tensile strength (UTS), can be significantly influenced by print parameters and environmental conditions.

An additional important consideration in the selection of filaments was their suitability for additive manufacturing and subsequent mycelial colonization. Polylactic acid (PLA) was chosen because it is one of the most widely used biopolymer filaments in fused filament fabrication, offering reliable printability, renewable feedstock origins, and biodegradability under industrial composting conditions [14, 15]. In addition, a PLA_Hemp composite filament was included in the study, as the incorporated hemp particles provide a lignocellulosic substrate that may facilitate mycelial colonization and growth [16]. No additional material characterization or toxicity testing was conducted in the present study.

### Additively manufactured specimens

Additive manufacturing was employed to produce standardized tensile specimens in order to investigate the influence of material composition and printing parameters on mechanical performance and fungal colonization behavior.

#### Printing method and equipment

Material Extrusion Additive Manufacturing (MEX AM) was applied to produce standardized tensile test specimens. This method was chosen due to its suitability for printing both neat PLA and particle-reinforced PLA_Hemp. The use of PLA_Hemp, a composite material containing natural fibers, can enhance mechanical properties through reinforcement mechanisms such as improved load transfer and crack resistance. Investigating the behavior of these materials during printing helps determine optimal printing parameters and hardware settings for high-quality prints [17].

**Fig. 1:**
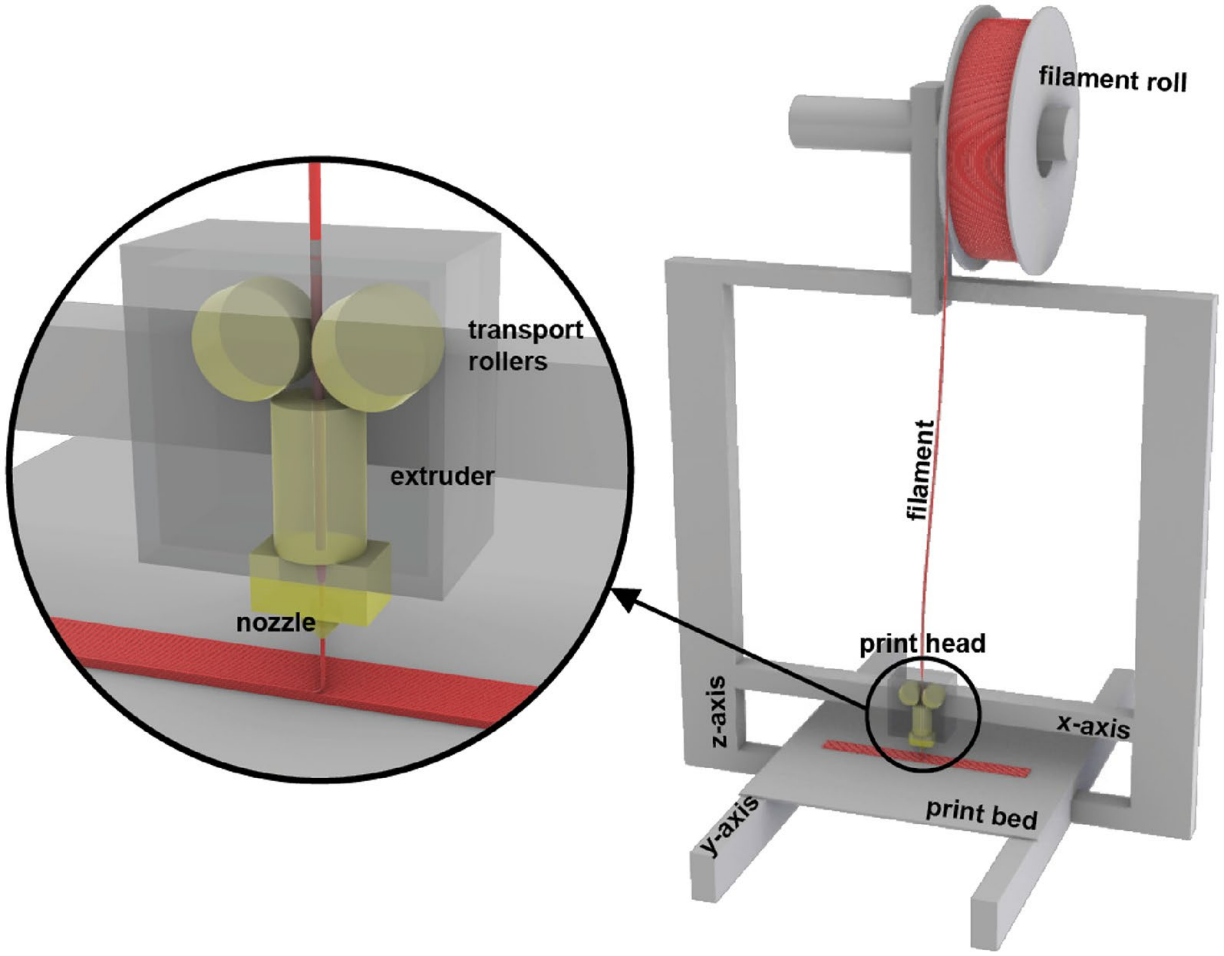
Schematic of MEX AM 3D-printing process [19]

In the MEX AM process, the print head operates on two of the three axes of the three-dimensional coordinate system. A Prusa i3 MK3S+ (Prusa Research a.s., Prague, Czech Republic) [18] was used for all experiments. The print head moves along the *x*- and *z*-axes, while movement along the *y*-axis is achieved by the print bed. The filament is transported through rollers into the heated extruder, melted, and deposited layer by layer onto the print bed. The process is illustrated in Fig. 1.

#### Specimen design

**Fig. 2:**
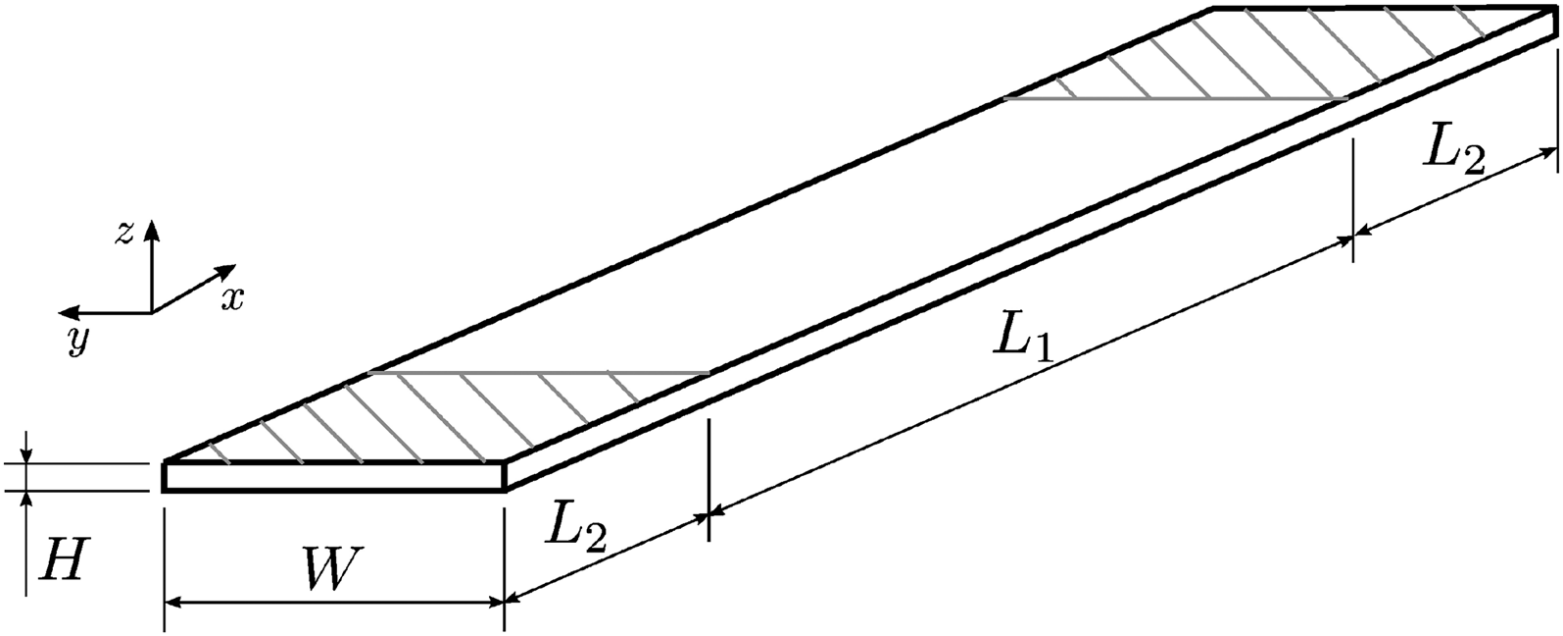
Sketch of the test specimens following ASTM D3039 [19]

**Fig. 3:**
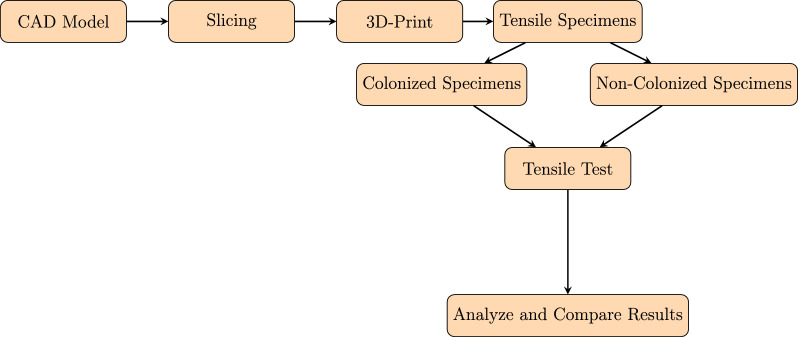
Schematic representation of the process workflow [22]

The specimens were designed following the tensile test standard ASTM D3039 [20]. Figure 2 shows the specimen geometry. The 3D model was created using Rhinoceros 3D (Rhino) (TLM, Inc., Seattle, WA, USA) [21] with dimensions $L_{1} = 120\, \textrm{mm}$, $L_{2} = 30\, \textrm{mm}$, $W = 15\, \textrm{mm}$, and $H = 2\, \textrm{mm}$. The design and fabrication workflow is schematically summarized in Fig. 3.

#### Printing patterns and orientations

**Fig. 4:**
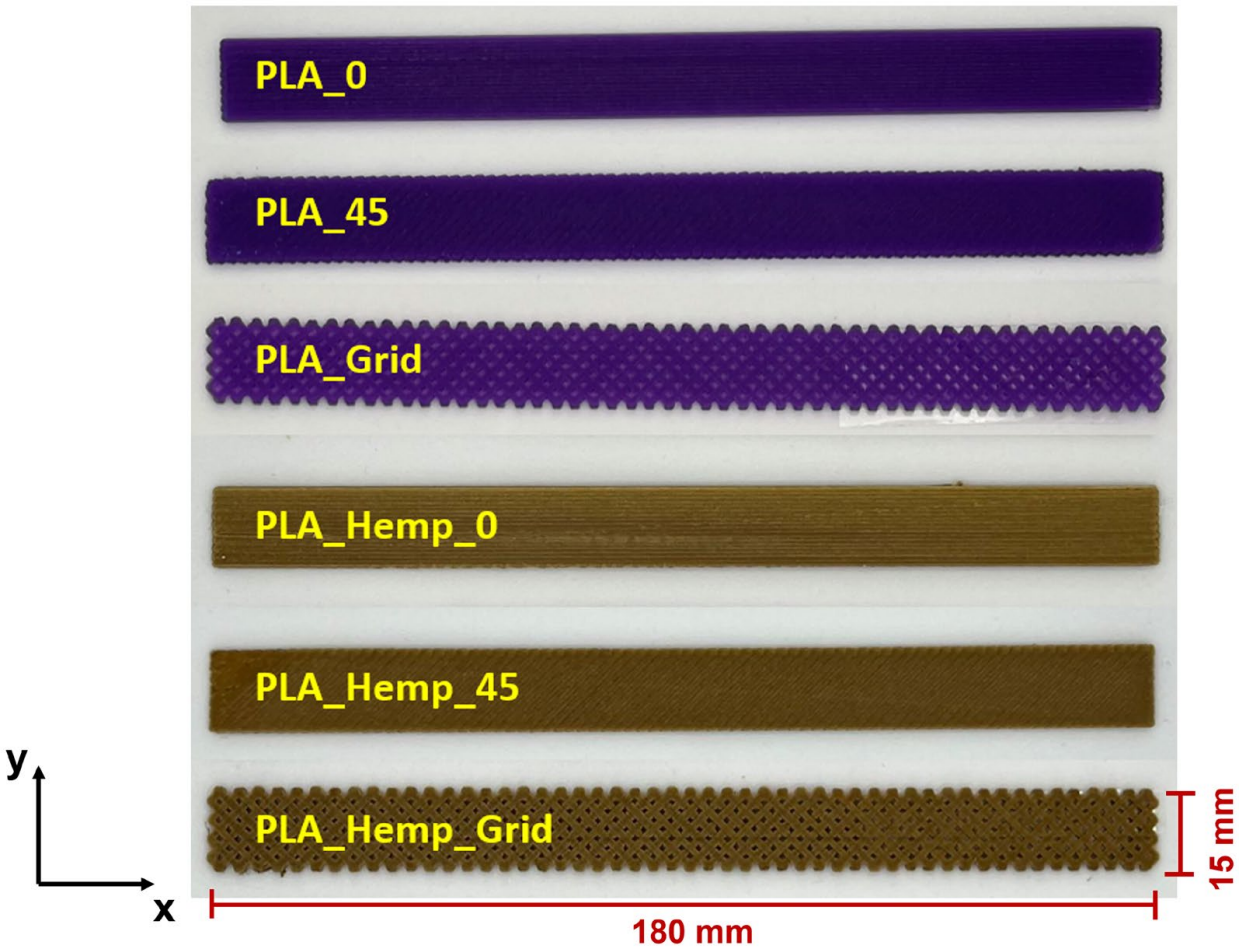
Overview of additively manufactured PLA and PLA_Hemp specimens with varying print pattern orientations (Pattern I, II, and III)

Three different infill orientations were investigated: linear (0°), diagonal (45°), and grid patterns. These orientations are crucial as they determine the anisotropic mechanical behavior of the specimens, influencing tensile strength, stiffness, and fracture strain. Table 2 summarizes the print path orientations, and Fig. 4 shows representative specimens.

**Table 2:** Print path orientation for different printing patterns

Print pattern	Orientation (degrees)	Pattern
Pattern I	0	Linear
Pattern II	45	Diagonal
Pattern III	Grid	Grid infill

The different infill orientations were selected not only to assess their mechanical effects but also to explore how mycelium colonization interacts with these internal structures. It is hypothesized that the infill pattern may facilitate or hinder hyphal attachment and growth, thereby affecting the composite’s mechanical performance [23–25].

#### Printing parameters

**Fig. 5:**
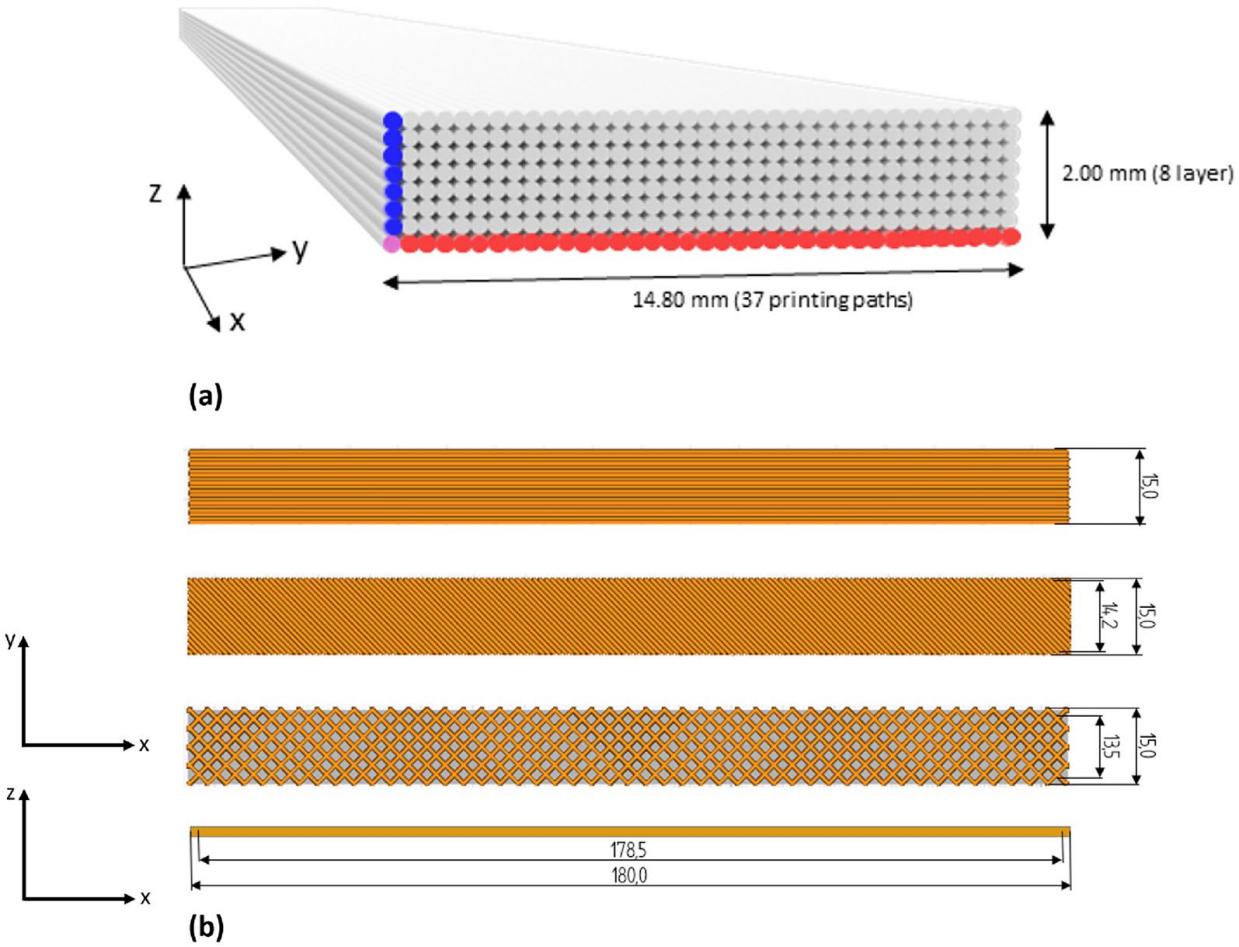
**a** Perspective sketch of the test specimen, showing layer structure and print patterns, and **b** three different print orientations (Pattern I, II, III)

Printing parameters were established using the open-source slicing software Ultimaker Cura (Version 5.2.1). Each 3D-printed layer had a height of 0.25 mm and a width of 0.4 mm. Given the specimen height of 2 mm and width of 15 mm, this resulted in 8 layers and 37 printing lines per layer (Fig. 5). The complete set of parameters is listed in Table 3.

**Table 3:** Printing parameters according to Ultimaker Cura for the tensile specimens. Differences in patterns are denoted with /

	MEX AM 3D printer
Printer	PRUSA i3 MK3S+
*Nozzle diameter*	0.8 mm
*Quality*	
*Layer height*	0.25 mm
Line width	0.38 mm
*Infill Parameters*	
Infill Density (Pattern I, II, III)	100% / 100% / 70%
*Infill Pattern*	Lines / Lines / Grid
Infill line directions	[90] / [−45] / –
*Material and temperature*	
Printing Temperature	190–205°C
*Bed Temperature*	65°C
*Speed and cooling*	
*Print Speed*	45 mm/s
Cooling	off

#### Post-processing and conditioning

Three specimens were printed simultaneously to analyze the effect of bed position on mechanical properties, as print-bed temperature gradients can induce internal stress and anisotropy [26, 27]. To ensure consistent conditions, new and dry PLA filaments were used. After printing, specimens were dried at 40°C for 6 hours to remove residual moisture. This step ensured environmental consistency, as moisture is known to affect PLA’s internal structure and mechanical behavior [28, 29].

### Experimental setup and testing procedure

**Fig. 6:**
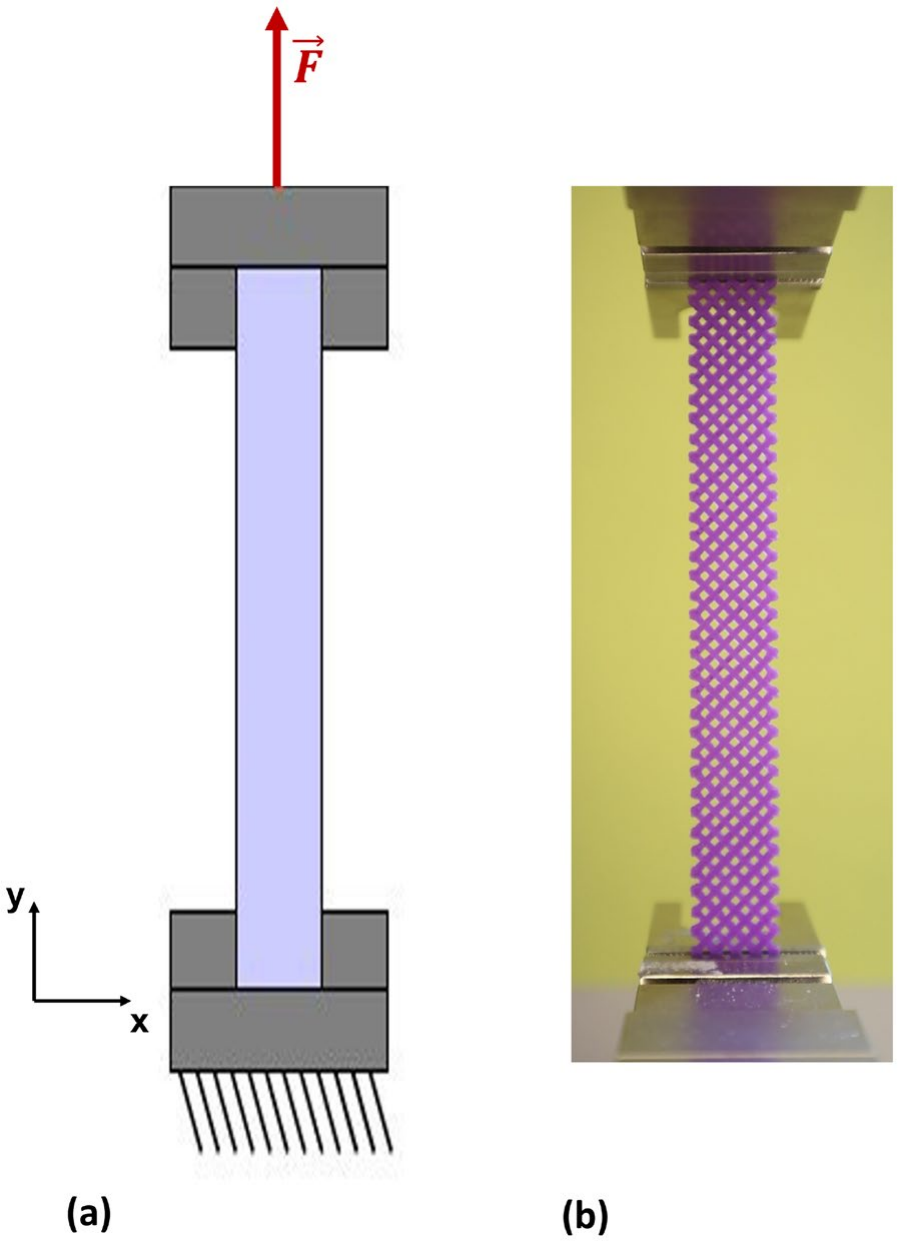
**a** Schematic representation of the tensile test and **b** experimental setup with the ZwickRoell Z2.5 testing machine

Material testing was performed using a ZwickRoell Z2.5 universal testing machine (GmbH & Co. KG). Figure 6 illustrates the setup employed for the tensile experiments. The machine provides a testing space depth of 105 mm along the *x*-axis and a crosshead travel range from 90 mm to 920 mm along the *y*-axis, allowing high flexibility for accommodating various specimen geometries. These geometrical constraints were carefully considered when selecting specimen dimensions to ensure compliance with standardized tensile testing protocols.

During testing, specimens were securely clamped using specialized grips to prevent slippage. The grips moved at a constant crosshead speed of 2 mm/min, ensuring uniform tensile loading. Specimens were loaded until failure, undergoing elongation and ultimately fracture. The tensile specimens were designed such that the maximum load capacity of the testing machine, 2.5 kN, was sufficient for all tested materials.

This testing setup enabled precise control of displacement, force, and speed, ensuring high data accuracy and repeatability, which is critical for reliable evaluation of mechanical properties. The recorded force-displacement data were subsequently used to calculate key material parameters, including Young’s modulus and ultimate tensile strength (UTS). These results are essential for the development and optimization of materials across a range of engineering applications.

A total of 21 experimental series were produced, each consisting of 10 specimens, resulting in an overall total of 210 specimens. For each series, 10 specimens were colonized with mycelium, while the remaining 10 were left untreated. For each print pattern, 10 specimens were exposed to mycelium colonization over a period of two weeks. After this period, the mycelium growth was halted, as explained in the following subsection. Subsequently, all specimens underwent tensile testing to determine their stress-strain behavior. Tensile tests were conducted under standardized conditions to ensure comparability of results.

Additionally, a time-dependence study was performed using specimens with Pattern I. Thirty specimens were prepared for each filament described in Sect. 2.1. For each material, 10 specimens were tested without mycelium colonization, 10 after two weeks of colonization, and 10 after four weeks of colonization.

Table 4 provides a comprehensive overview of all specimens produced and utilized in this study, including specimen designation, material composition, print pattern, and mycelium processing conditions.

**Table 4:** Overview of specimen series

Specimen	Number	Material	Print pattern	Mycelium processing	
				Applied	Duration
PLA_0	10	PLA 1	Pattern I	No	–
PLA_45	10	PLA 1	Pattern II	No	–
PLA_Grid	10	PLA 1	Pattern III	No	–
PLA_WM_0	10	PLA 1	Pattern I	Yes	2 weeks
PLA_WM_45	10	PLA 1	Pattern II	Yes	2 weeks
PLA_WM_Grid	10	PLA 1	Pattern III	Yes	2 weeks
PLA_Hemp_0	10	PLA_Hemp	Pattern I	No	–
PLA_Hemp_45	10	PLA_Hemp	Pattern II	No	–
PLA_Hemp_Grid	10	PLA_Hemp	Pattern III	No	–
PLA_Hemp_WM_0	10	PLA_Hemp	Pattern I	Yes	2 weeks
PLA_Hemp_WM_45	10	PLA_Hemp	Pattern II	Yes	2 weeks
PLA_Hemp_WM_Grid	10	PLA_Hemp	Pattern III	Yes	2 weeks
PLA_0	20	PLA 1 and PLA 2	Pattern I	No	–
PLA_WM_2W_0	20	PLA 1 and PLA 2	Pattern I	Yes	2 weeks
PLA_WM_4W_0	20	PLA 1 and PLA 2	Pattern I	Yes	4 weeks
PLA_Hemp_0	10	PLA_Hemp	Pattern I	No	–
PLA_Hemp_2W_0	10	PLA_Hemp	Pattern I	Yes	2 weeks
PLA_Hemp_4W_0	10	PLA_Hemp	Pattern I	Yes	4 weeks

Following specimen fabrication and treatment, detailed descriptions of the initial prints, experimental trials, and main testing sequences were provided. This approach enabled a comprehensive evaluation of the impact of mycelium colonization on the mechanical properties of the specimens. The distinction between colonized and non-colonized specimens was essential to assess the effects of mycelium growth on tensile strength and other mechanical characteristics [19].

### Mycelium processing

After producing the tensile specimens with three different patterns and with the aforementioned two filament materials, 10 tensile specimens from each test series were used to investigate the effect of fungal colonization on the 3D-printed specimens.

For this purpose, a solid plant substrate of hemp shives, which was intensively overgrown with mycelium of the tinder fungus *Fomes fomentarius*, was prepared in which the specimens could be embedded.

The biotechnological production of materials using pure cultures of microorganisms requires special working procedures to provide the desired organism with optimal growth conditions while preventing contamination from foreign microorganisms such as mold spores. Such contamination can compromise material stability and homogeneity and may pose health hazards.

All cultivation work was therefore carried out using appropriate sterile techniques in microbiological laboratories. Work was performed under a sterile bench whenever possible, and a laminar flow cabinet was used for later steps involving large culture vessels. All required materials and tools were autoclaved when possible. If this was not an option, intensive wiping with 70% EtOH was carried out.

All incubation steps were carried out in the dark at approximately 25°C. Solid substrates were sufficiently hydrated based on experience from previous studies.

**Fig. 7:**
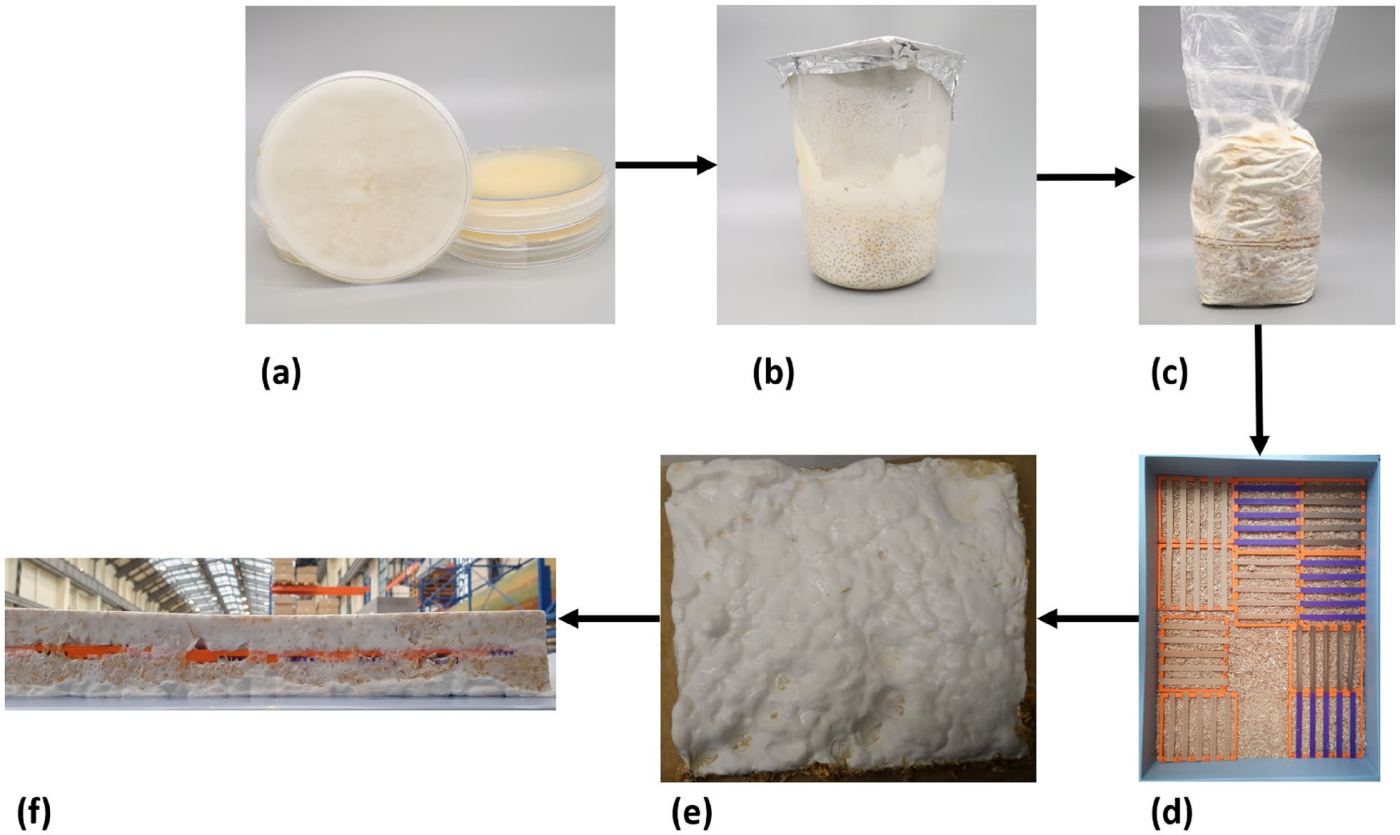
Laboratory manufacturing process for *Fomes fomentarius* composite materials. **a** agar plate pure culture, used to inoculate: **b** sterilized millet overgrown with *Fomes fomentarius* mycelium, used to inoculate: **c** hemp shive culture in cultivation bag. After incubation the material is shredded and used for embedding of specimen carriers as displayed with only bottom layer in (**d**). After adding the top layer another incubation step leads to formation of a completely overgrown solid mycelium-substrate-specimen block (**e**) and (**f**). Drying and specimen harvesting are the following steps

The cultivation of *Fomes fomentarius* strain PaPF11 on hemp shives substrate and the subsequent composite production followed a previously published procedure [7, 9]. These steps are briefly outlined here and illustrated in Fig. 7.

#### Agar plate pure culture

A pure culture of *Fomes fomentarius* strain PaPF11 was isolated from a fruiting body specimen as described in [7]. The resulting strain was then repeatedly transferred to new agar plates, which after incubation were stored at 5°C. Additionally the strain was backed up using cryopreservation at -80°C. This set of methods is essential for strain maintenance. Two-week-old fully overgrown malt extract agar plates were used as inoculum for the next step.

#### Grain spawn on millet

Brown millet, pre-treated by autoclaving, was inoculated and incubated for two weeks, resulting in a fully mycelium-covered volume. This step expands the mycelium biomass and allows metabolic adaptation from a sugar-based medium to more complex organic substrates, preparing it for the main substrate.

#### Cultivation on hemp shives

The millet grain spawn was used to inoculate hemp shives, ensuring rapid and even colonization as each grain serves as a growth initiation point. Hemp shives, an agricultural by-product, offer a high-value material application due to their wood-like structure and chemical composition, rich in lignin. Although *Fomes fomentarius* naturally grows on deciduous trees, it successfully colonized the hemp substrate in this controlled setting. The mixture was incubated for two weeks, with a manual break-up and remixing after one week. By the end of incubation, the hemp shives were uniformly colonized and interwoven by a dense network of fungal hyphae [9].

#### Shredding

Shredding was carried out using a commercial shredder, reducing the material to small particles comparable in size to the original hemp shives. These particles were then used for the final composite shaping using molds. This step temporarily disrupts the mycelial connections, but rapid regrowth occurs in the following phase.

#### Pre-treatment of specimens for sterilization

To ensure the sterility of the 3D-printed specimens before embedding them in the fungal mycelium composite, different sterilization methods were evaluated. The tested approaches included autoclaving, ethanol wiping, UV exposure, and no treatment as a control.

Autoclaving was not feasible due to the heat sensitivity of the 3D-printed PLA and PLA_Hemp specimens, which could lead to deformation or mechanical property alterations [30, 31]. PLA-based materials are known to degrade at elevated temperatures, making alternative sterilization techniques necessary. Instead, ethanol (EtOH) was selected as the primary sterilization method due to its effectiveness in deactivating microbial contaminants while preserving the integrity of the specimens [32]. The specimens were thoroughly wiped with 70% EtOH, ensuring an exposure time of at least 30–60 seconds per surface , a duration recommended for effective microbial and fungal disinfection in laboratory settings.

Additionally, specimen holders were subjected to UV treatment inside a laminar flow cabinet to further minimize contamination risks. UV exposure has been widely used for surface sterilization and has been shown to effectively reduce microbial contamination [1]. The UV exposure was applied for 20 minutes per side [33]. However, direct UV exposure was avoided for the specimens themselves to prevent potential material degradation, which has been reported in previous studies [34, 35].

These pre-treatment steps ensured a controlled and contamination-free environment for embedding the PLA and PLA_Hemp specimens in the fungal mycelium composite. Ethanol pretreatment was considered sufficient to achieve the required germ reduction for embedding, as complete sterility is not essential once the substrate is fully colonized by *F. fomentarius*. At this stage, well-established mycelial networks generally suppress competing microorganisms, with only sporadic molds or spore-forming bacteria occasionally observed. Conventional CFU-based assays were therefore not applied, as they would predominantly detect non-critical background organisms and provide limited insight into relevant contamination risks. A more informative approach would involve targeted molecular quantification (e.g., genus-specific qPCR), which, however, lies beyond the scope of the present study and will be pursued in future work. To exclude inhibitory effects from residual ethanol, treated specimens were allowed to dry completely under a laminar flow cabinet prior to embedding. Consistent with previous long-term cultivation experience, no adverse effects on mycelial colonization attributable to ethanol residues were observed.

#### Embedding of specimens in the mycelium composite

After shredding of the hemp shives colonized with mycelium, the 3D-printed tensile specimens were embedded in the fungal mycelium material. To account for purely environmental influences, additional non-colonized specimens were incubated under identical conditions. These control specimens were placed in sterilized containers, with one group fixed with a lid (fixed) and another group left without a lid (unfixed), in order to capture potential passive effects such as moisture absorption or material relaxation. Box-shaped growth chambers were used for embedding. A bottom layer of shredded mycelium-hemp composite was added, followed by placement of the 3D-printed specimens in specimen holders to ensure uniform orientation and spacing. Another layer of shredded composite was placed on top. Light shaking and pressure compaction ensured close and even contact between the mycelium and specimens, eliminating hollow spaces. The assembly was then incubated for two to five weeks, allowing mycelium to grow out and form a cohesive structure around the embedded specimens. Continuous sterility monitoring during the incubation period (e.g., scheduled swab or imprint cultures) was not implemented, as the experimental setup was designed to reflect conditions relevant for industrial-scale production, where complete sterility is not practicable. Instead, emphasis was placed on maintaining reduced-germ conditions that reliably enabled uniform colonization of the specimens and the formation of cohesive composite materials.

#### Drying and harvesting of mycelized specimens

After the incubation period, aseptic handling was no longer required. At this stage, the mycelium-based composite had developed into a cohesive material but still contained high moisture levels [36]. To ensure dimensional stability and halt further biological activity, the composite blocks were dried in a laboratory oven at 55°C. This thermal treatment reduced the moisture content from over 50 % to a stable range of approximately 10–25 %, in line with established procedures for mycelium composite processing [36, 37]. The drying process resulted in a lightweight, rigid, and biologically inactive material suitable for further mechanical testing.

Following drying, the embedded specimens were harvested. The upper layer of the composite was carefully removed to expose the specimens, which were then extracted. Residual substrate or overgrown mycelium was trimmed to conform to the defined specimen geometry [9]. The specimens were stored under controlled conditions until mechanical testing. Figure 9 illustrates specimens before and after mycelium extraction. After extraction, the specimens were vacuum-sealed for preservation before testing.

## Results

Macroscopic and microscopic analyses of mycelium colonization are first described, followed by the mechanical response of 3D-printed PLA and PLA_Hemp specimens under different processing and exposure conditions.

### Macroscopic observations of mycelium colonization

**Fig. 8:**
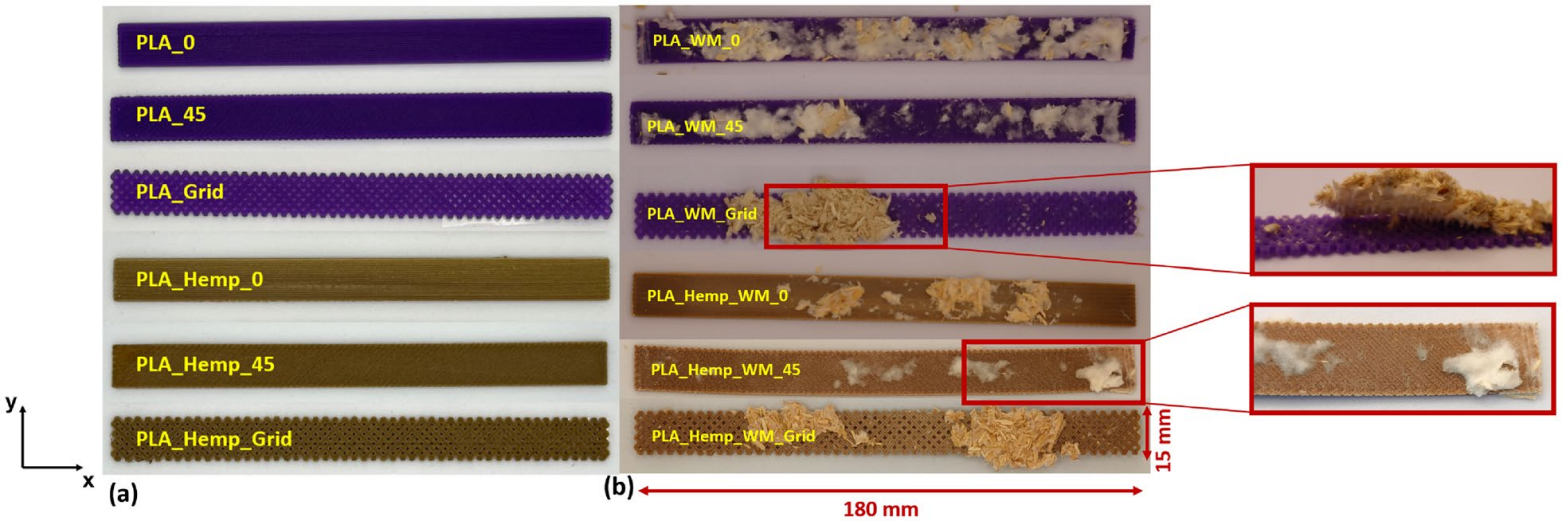
Overview of additively manufactured PLA and PLA_Hemp specimens with varying print pattern orientations (Pattern I, II, and III), shown **a** prior to and **b** following mycelium colonization

Based on initial evaluations of specimen production, handling, and preliminary colonization tests, the main study was organized into two parts. The first part focused on the effect of material, comparing mechanical performance across all specimen types. The second part examined the influence of print pattern. An overview of the additively manufactured specimens is provided in Fig. 8.

**Fig. 9:**
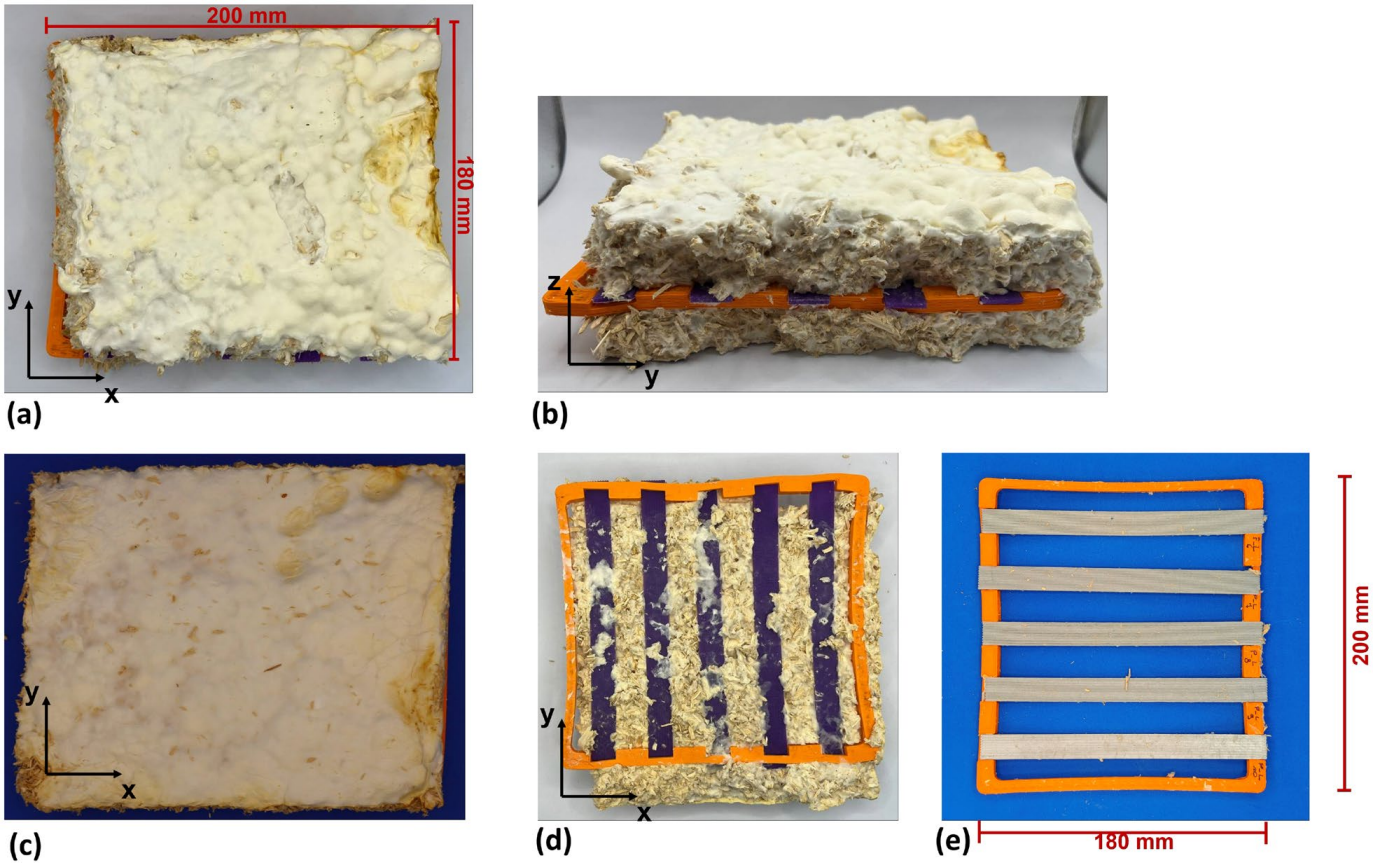
Mycelium-colonized specimens: **a** and **d** top view of specimens over three weeks and six days, **b** front view over the same period, **c** and **e** top view after two weeks

**Fig. 10:**
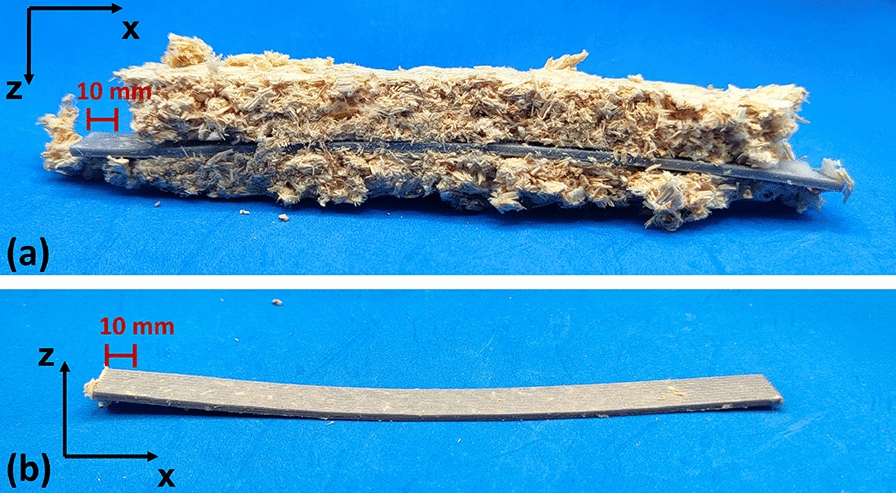
Specimens colonized by mycelium over three weeks and six days, showing hardened mycelium and slight bending: **a** side view with mycelium and **b** side view after removal of mycelium

Specimens colonized over two weeks and over three weeks and six days are presented in Figs. 9 and 10, respectively. In the two-week colonized specimens, the mycelium penetrated internal voids and overgrew the surface of Pattern III specimens, particularly for PLA_Hemp, while a lighter coloration was observed due to biochemical effects on the material surfaces. For the specimens colonized over three weeks and six days, the mycelium appeared darker and exhibited a hardened texture, and slight bending of the specimens was evident. These observations indicate that prolonged mycelium colonization intensifies changes in color and surface texture and can also affect the structural integrity of the specimens.

### Mechanical properties

Uniaxial tensile test results are presented with an emphasis on the effects of mycelium colonization, printing pattern, specimen position on the build platform, and colonization duration. Mechanical performance was evaluated using mean values, standard deviations, and 95% confidence intervals [38]. All specimens were included in the analysis, as none exhibited deviations that would compromise statistical relevance [38, 39]. These results provide insights into the interplay between material composition, print pattern orientation, and duration of mycelium colonization on tensile performance. The observations systematically quantify how mycelial growth influences both mechanical integrity and visible surface characteristics, demonstrating the effects of biological integration on additively manufactured polymer composites.

#### Determination of stiffness and strength

The stiffness in the loading direction (Young’s modulus) and the ultimate tensile strength (UTS) of all specimen types were determined in accordance with ASTM D 39039 [38], as illustrated in Figure ??. The Young’s modulus was calculated at strain points of 0.001 and 0.003 to ensure reproducible evaluation of the elastic region. A comprehensive overview of both stiffness and strength parameters, including mean values and standard deviations (SD), is provided in Table 5.

**Table 5:** Overview of the mechanical properties (Young’s modulus and ultimate tensile strength, UTS) of additively manufactured PLA 1 and PLA_Hemp specimens with different print patterns. Values are given as mean ± standard deviation (SD)

Specimen	Young’s modulus		Ultimate tensile strength (UTS)	
	Mean	SD	Mean	SD
	(N/mm^2^)	(N/mm^2^)	(N/mm^2^)	(N/mm^2^)
PLA_0	2867.36	15.63	62.62	1.66
PLA_45	2840.82	15.25	58.07	0.43
PLA_Grid	665.69	7.24	10.32	0.14
PLA_WM_0	2951.57	10.83	63.43	0.31
PLA_WM_45	2879.35	6.09	57.17	0.28
PLA_WM_Grid	761.78	6.82	11.29	0.13
PLA_Hemp_0	3072.42	14.55	43.25	0.28
PLA_Hemp_45	2814.35	10.46	36.11	0.09
PLA_Hemp_Grid	592.64	4.57	7.51	0.06
PLA_Hemp_WM_0	3003.71	25.59	41.10	0.27
PLA_Hemp_WM_45	2773.51	14.17	34.70	0.27
PLA_Hemp_WM_Grid	564.98	14.86	6.94	0.16

#### Analysis of variance (ANOVA)

**Fig. 11:**
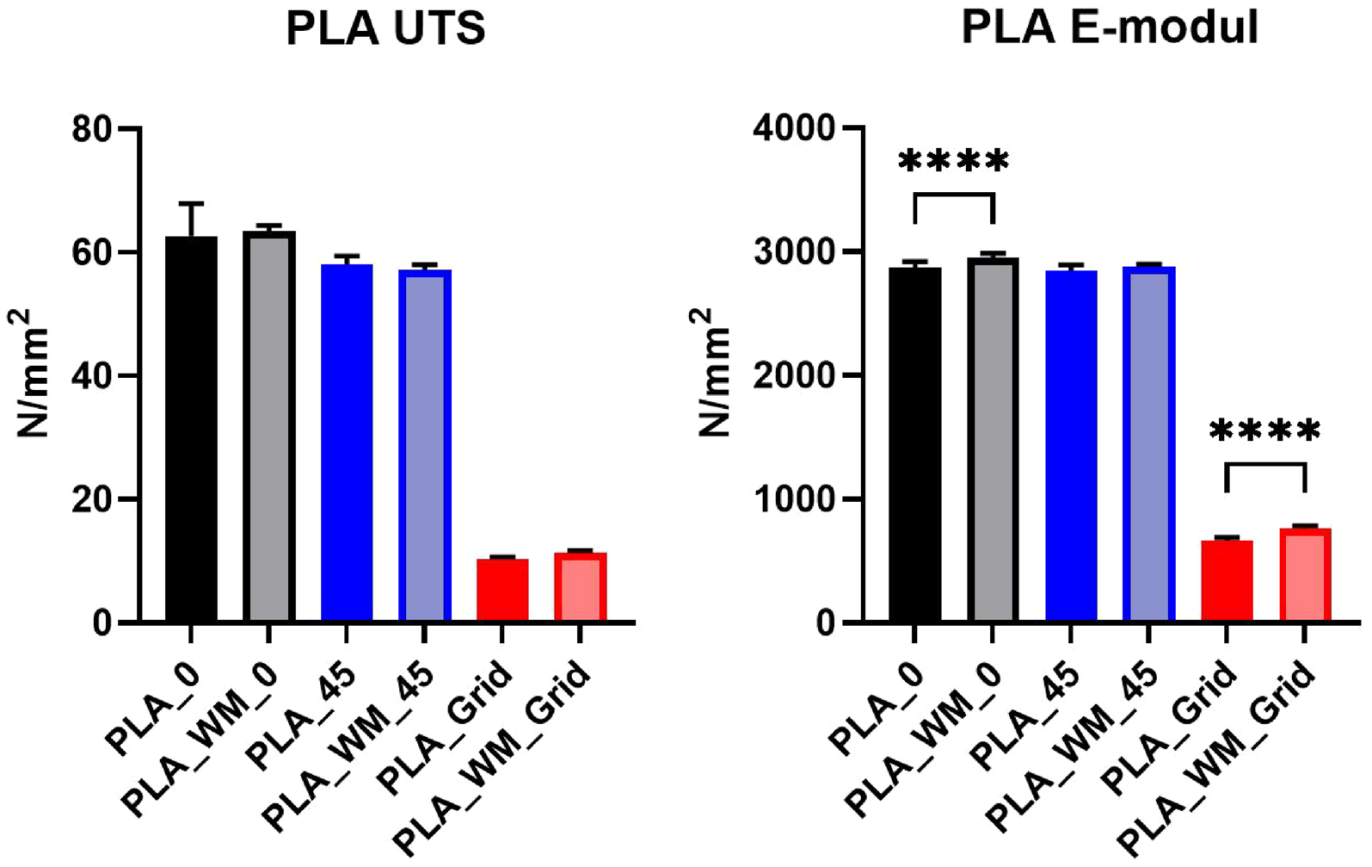
Ultimate tensile strength (left) and mean Young’s modulus (right) of PLA specimens. Grid-pattern specimens exhibited dramatically reduced performance (****p < 0.0001). A significant difference was also observed between PLA_0 and PLA_WM_0 in stiffness (****p < 0.0001)

**Fig. 12:**
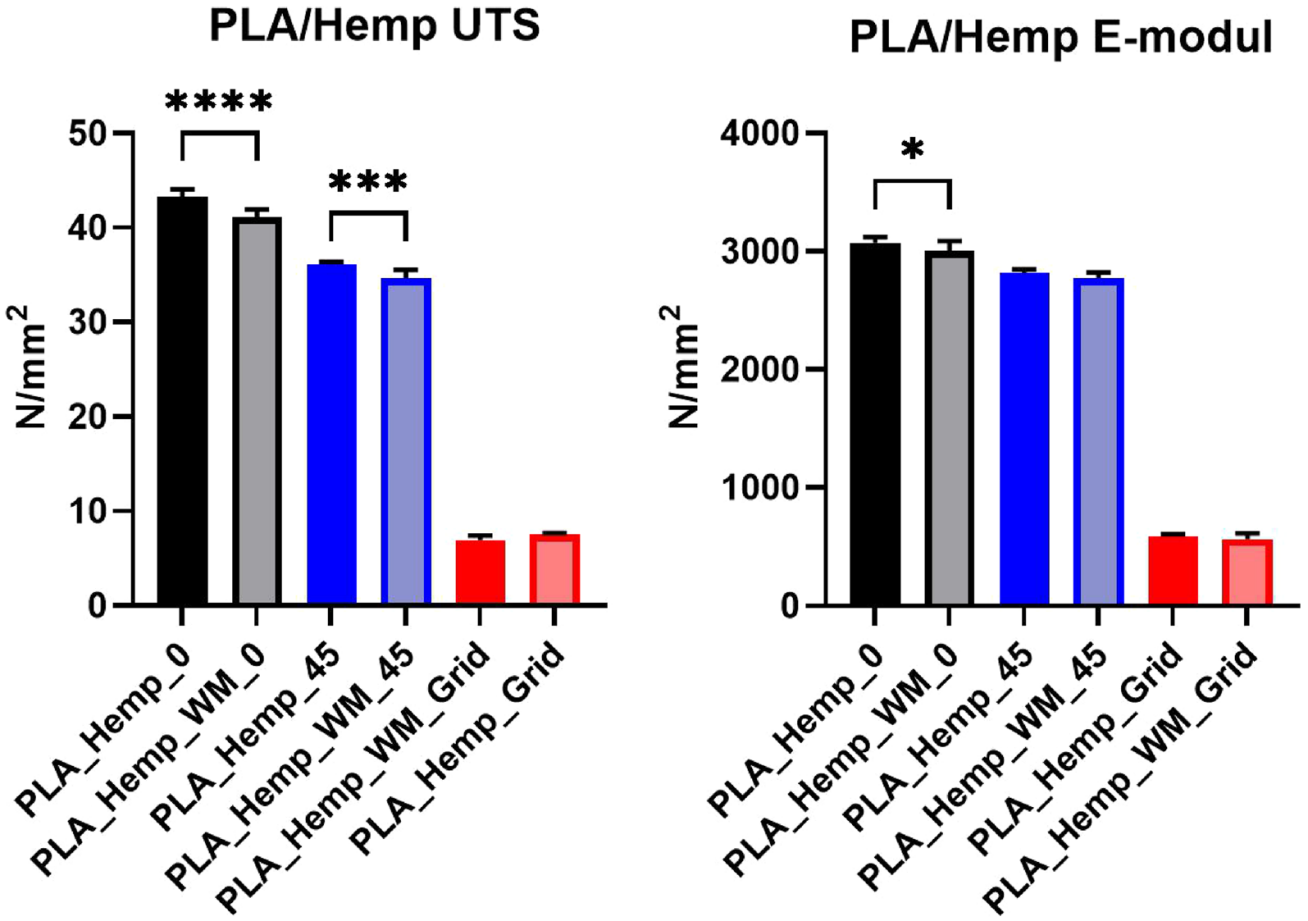
Ultimate tensile strength (left) and mean Young’s modulus (right) of PLA_Hemp specimens. Orientation strongly influenced the UTS, with significant reductions at 0° (****p < 0.0001) and 45° (***p < 0.001). Stiffness was moderately reduced in hemp-reinforced specimens, significant at *p < 0.05

To statistically assess differences among the specimen groups, a one-way analysis of variance (ANOVA) followed by Tukey’s post-hoc test was performed [40]. The statistical analysis was conducted using GraphPad Prism (Version 10.6.1) [41]. Figures 11 and 12 present the ultimate tensile strength (UTS, left) and Young’s modulus (right) for PLA and PLA_Hemp specimens. Statistical significance is indicated as follows: *p < 0.05, **p < 0.01, ***p < 0.001, ****p < 0.0001, and ns = not significant.

#### Effect of pre-treatment on mechanical properties

**Fig. 13:**
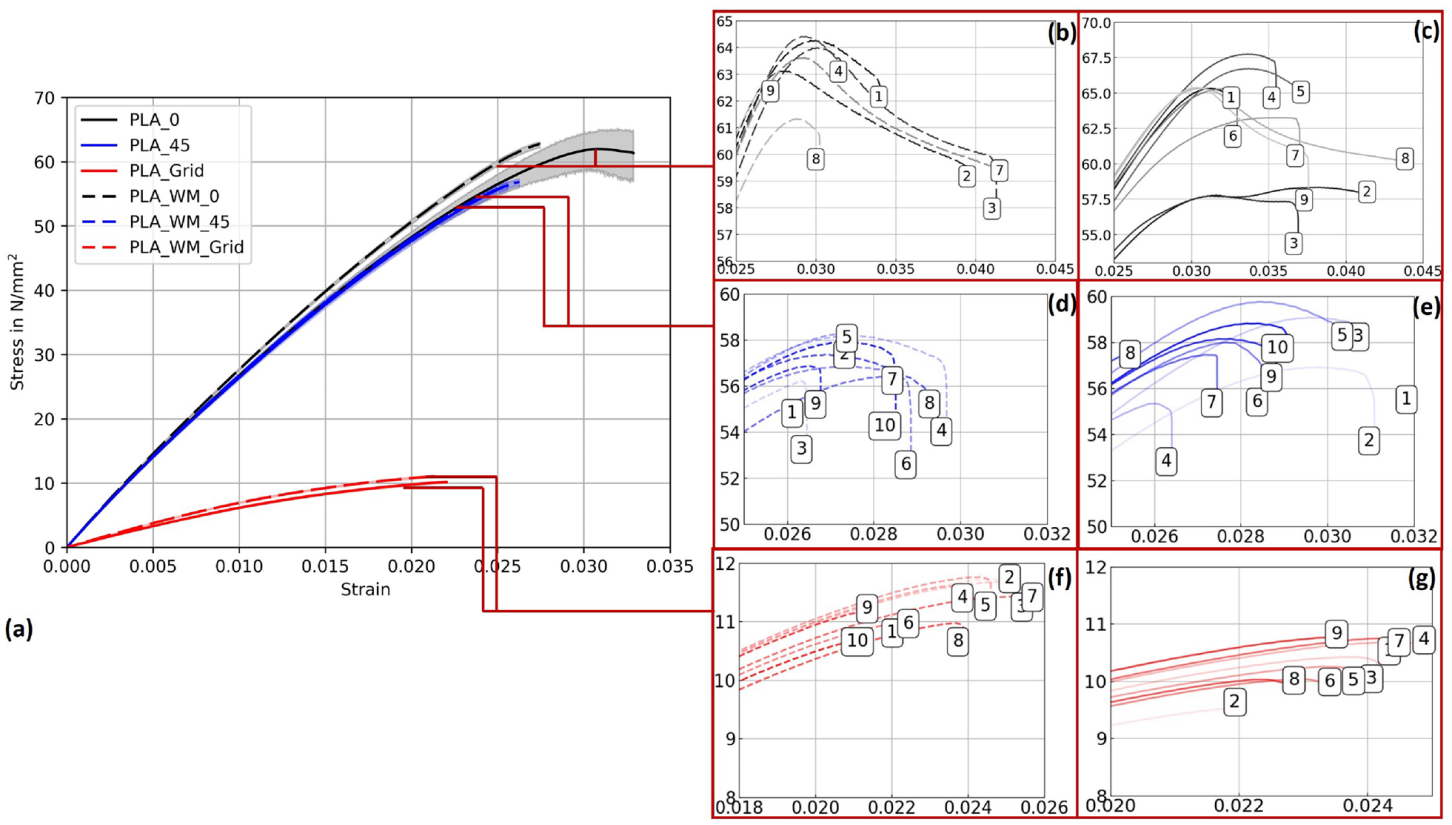
**a** Mean stress-strain diagram comparing the print pattern of PLA 1 specimens for Pattern I, Pattern II, and Pattern III. **b** Individual stress-strain curves for PLA 1 specimens with Pattern I after 2 weeks of mycelium colonization. **c** Individual stress-strain curves for PLA 1 specimens with Pattern I. **d** Individual stress-strain curves for PLA 1 specimens with Pattern II after 2 weeks of mycelium colonization. **e** Individual stress-strain curves for PLA 1 specimens with Pattern II. **f** Individual stress-strain curves for PLA 1 specimens with Pattern III after 2 weeks of mycelium colonization. **g** Individual stress-strain curves for PLA 1 specimens with Pattern III

**Fig. 14:**
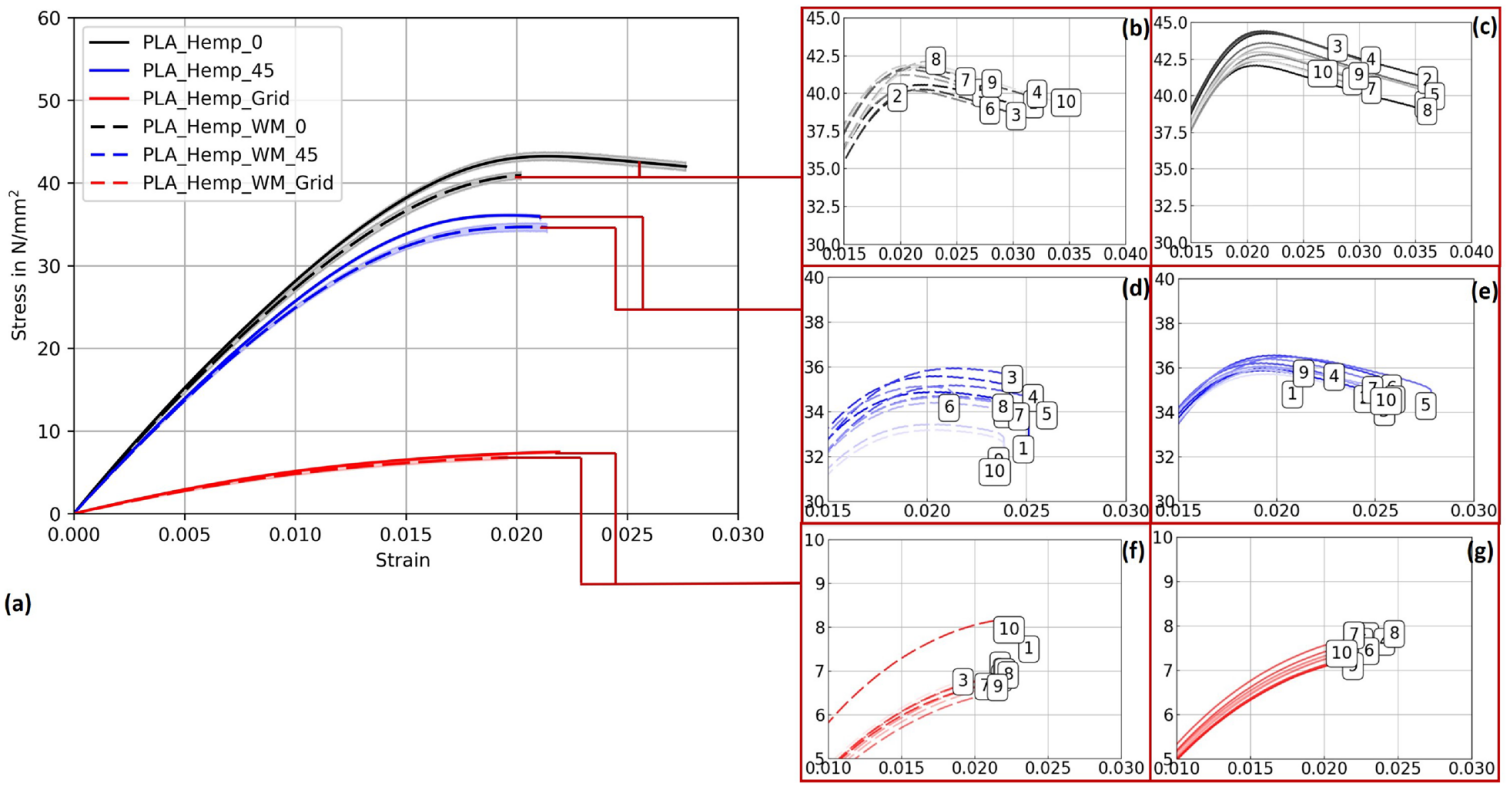
**a** Mean stress-strain diagram comparing the print pattern of PLA_Hemp specimens for Pattern I, Pattern II, and Pattern III. **b** Individual stress-strain curves for PLA_Hemp specimens with Pattern I. **c** Individual stress-strain curves for PLA_Hemp specimens with Pattern I after 2 weeks of mycelium colonization. **d** Individual stress-strain curves for PLA_Hemp specimens with Pattern II. **e** Individual stress-strain curves for PLA_Hemp specimens with Pattern II after 2 weeks of mycelium colonization. **f** Individual stress-strain curves for PLA_Hemp specimens with Pattern III. **g** Individual stress-strain curves for PLA_Hemp specimens with Pattern III after 2 weeks of mycelium colonization

To assess the influence of different pre-treatment methods on mechanical performance, tensile tests were conducted. At least one treatment was required to avoid microbial contamination and enable undisturbed mycelial colonization. The stress-strain curves for PLA_Hemp and PLA 1 specimens are shown in Figs. 14 and 13, respectively.

**Table 6:** Young’s modulus values of additively manufactured PLA 1 and PLA_Hemp specimens subjected to different pre-treatments. The percentage change is relative to the untreated specimens

Specimen	Young’s modulus (N/mm^2^)	Change (%)
PLA_Untreated	2797.25	–
PLA_Autoclaved	3418.33	+22.19
PLA_Ethanol	2805.89	+0.31
PLA_UV	2834.23	+1.32
PLA_Hemp_Untreated	2856.48	–
PLA_Hemp_Autoclaved	3049.78	+6.77
PLA_Hemp_Ethanol	2863.33	+0.24
PLA_Hemp_UV	2868.80	+0.43

As shown in Table 6, the choice of sterilization method significantly affects the stiffness of both PLA 1 and PLA_Hemp specimens. For PLA 1, untreated specimens exhibited a Young’s modulus of 2797.25 N/mm^2^. Autoclaving led to the strongest increase, raising the modulus to 3418.33 N/mm^2^ (+22.19%). A similar but less pronounced effect was observed for PLA_Hemp, where autoclaving increased the modulus from 2856.48 to 3049.78 N/mm^2^ (+6.77%). This indicates that thermal treatment at elevated temperature and pressure promotes polymer chain rearrangement, thereby increasing stiffness. However, such changes are often accompanied by embrittlement, which can reduce ductility and limit the material’s usability in applications requiring flexibility [42, 43].

In contrast, ethanol sterilization caused only negligible changes in stiffness: PLA 1 increased slightly to 2805.89 N/mm^2^ (+0.31%), and PLA_Hemp to 2863.33 N/mm^2^ (+0.24%). This demonstrates that ethanol effectively sterilizes without compromising the mechanical properties, aligning with earlier findings that identify ethanol as a preferred sterilization method for biopolymers [32, 1].

UV treatment showed minor increases as well: PLA 1 reached 2834.23 N/mm^2^ (+1.32%), and PLA_Hemp 2868.80 N/mm^2^ (+0.43%). These small changes are likely attributable to surface effects caused by photodegradation [44]. While the immediate impact is limited, prolonged UV exposure may lead to polymer degradation, reducing long-term stability.

Overall, these results clearly indicate that ethanol sterilization is the most suitable method, as it ensures microbial decontamination while maintaining the mechanical integrity of both PLA 1 and PLA_Hemp specimens. Autoclaving, although increasing stiffness, significantly alters the polymer structure and may cause embrittlement, whereas UV treatment poses risks of surface degradation over time.

#### PLA 1 specimens

Figure 13 presents the stress–strain behavior of PLA 1 specimens across three printing patterns, with 95% confidence intervals. While mycelium colonization had only a moderate influence on the mechanical performance of PLA, systematic trends were observed across all pattern orientations. The corresponding mechanical properties, including Young’s modulus and ultimate tensile strength (UTS) with their respective standard deviations (SD), are summarized in Table 5.

For Pattern I, mycelium-colonized specimens (PLA_WM_0) exhibited a slight increase in stiffness compared to their non-colonized counterparts (PLA_0). The Young’s modulus increased by 2.9%, from 2867.36 N/mm^2^ to 2951.57 N/mm^2^. The ultimate tensile strength also increased slightly, from 62.62 N/mm^2^ to 63.43 N/mm^2^, indicating that colonization had no detrimental effect on tensile strength.

In Pattern II specimens, the Young’s modulus showed a marginal increase from 2840.82 N/mm^2^ (PLA_45) to 2879.35 N/mm^2^ (PLA_WM_45), corresponding to a gain of 1.3%. The ultimate tensile strength decreased slightly, from 58.07 N/mm^2^ to 57.17 N/mm^2^, suggesting that the mycelium colonization had only a negligible impact on tensile performance in this orientation.

The most pronounced differences appeared in Pattern III specimens. The Young’s modulus increased significantly by 14.4%, from 665.69 N/mm^2^ (PLA_Grid) to 761.78 N/mm^2^ (PLA_WM_Grid). The ultimate tensile strength also improved slightly, from 10.32 N/mm^2^ to 11.29 N/mm^2^. This notable enhancement in both stiffness and strength suggests that the porous grid structure enabled more effective mycelial integration into the material’s internal geometry, potentially reinforcing load-bearing zones.

#### PLA_Hemp specimens

Figure 14 illustrates the stress-strain curves for specimens 3D-printed at Pattern I, Pattern II, and Pattern III, providing insights into their corresponding mechanical strengths and behaviors. The accompanying Young’s modulus values, detailed in Table ??, reveal significant variations among patterns as well as between specimens with and without mycelium growth. Contrary to PLA 1 specimens, mycelium-inoculated PLA_Hemp specimens (PLA_Hemp_WM_0) exhibited a lower maximum tensile stress compared to non-inoculated specimens (PLA_Hemp_0), indicating a degradation in mechanical integrity with a Young’s modulus decrease of approximately 2.3%, from 3072.42 N/mm^2^ to 3003.71 N/mm^2^. Similarly, PLA_Hemp specimens with a Pattern II showed a decrease from 2814.35 N/mm^2^ (PLA_Hemp_45) to 2773.51 N/mm^2^ (PLA_Hemp_WM_45), corresponding to a loss of about 1.5%. Pattern III PLA_Hemp specimens also demonstrated poorer mechanical properties after colonization, with Young’s modulus decreasing by 4.7%, from 592.64 N/mm^2^ (PLA_Hemp_Grid) to 564.98 N/mm^2^ (PLA_Hemp_WM_Grid).

### Effect of mycelium growth time on the mechanical properties

For the time-series tensile tests, pattern I (linear, 100% infill) was deliberately chosen, as it represents a generic layer configuration that provides the most consistent mechanical response under uniaxial tension. Specimens with fully solid cross-sections are expected to sustain higher tensile loads, and to minimize stress concentrations and structural artifacts, in contrast to porous architectures such as Pattern III (grid, 70% infill), which have been shown in prior studies to reduce mechanical strength and increase variability [45, 46]. The primary objective of these experiments was to investigate how the duration of mycelial colonization affects the mechanical properties under conditions where the base material is fully loaded, without the effects of infill geometry distorting the results. A solid cross-section (corresponding to 100% infill in additive manufacturing terminology) was therefore employed, allowing for a clear detection of changes in Young’s modulus attributable to mycelial growth and ensuring that observed variations are caused by biological effects rather than structural heterogeneity.

**Fig. 15:**
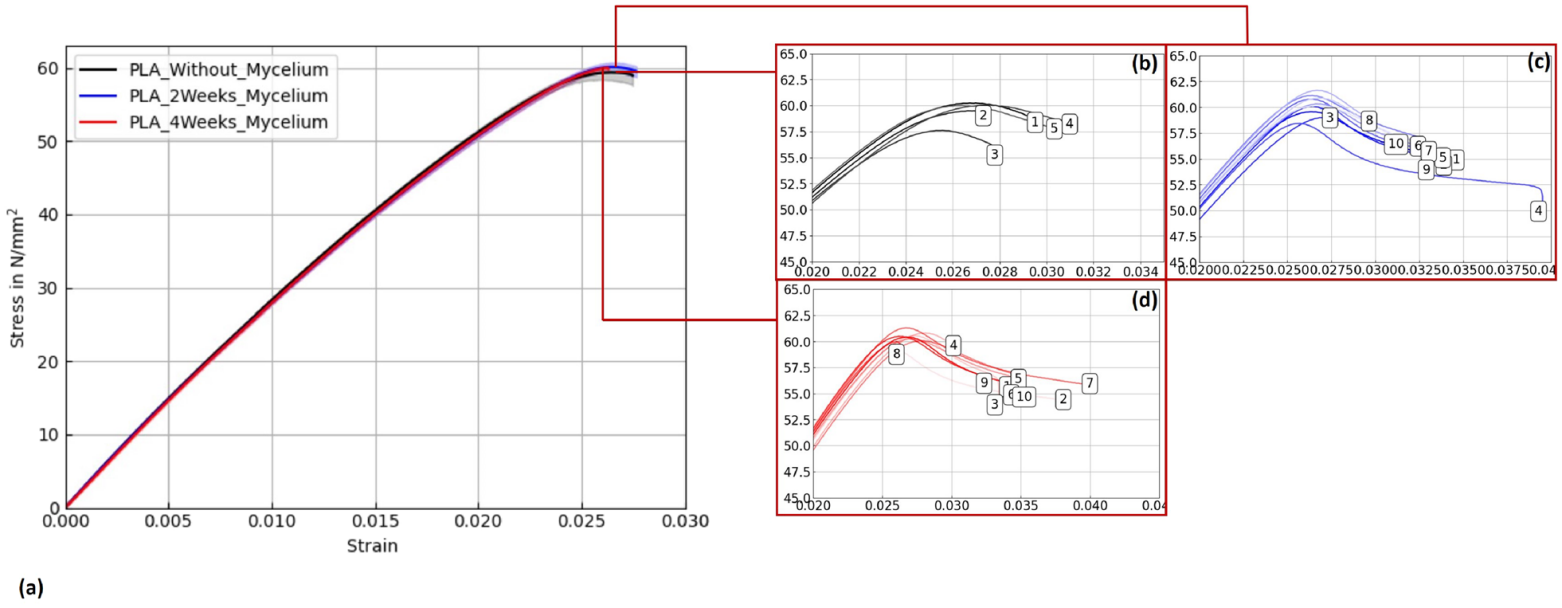
**a** Mean stress-strain diagram showing the effect of mycelium growth time on PLA 2 specimens (Prusament PLA Galaxy Silver, Pattern I). **b**–**d** Individual curves for untreated, 2-week, and 4-week colonized specimens

**Fig. 16:**
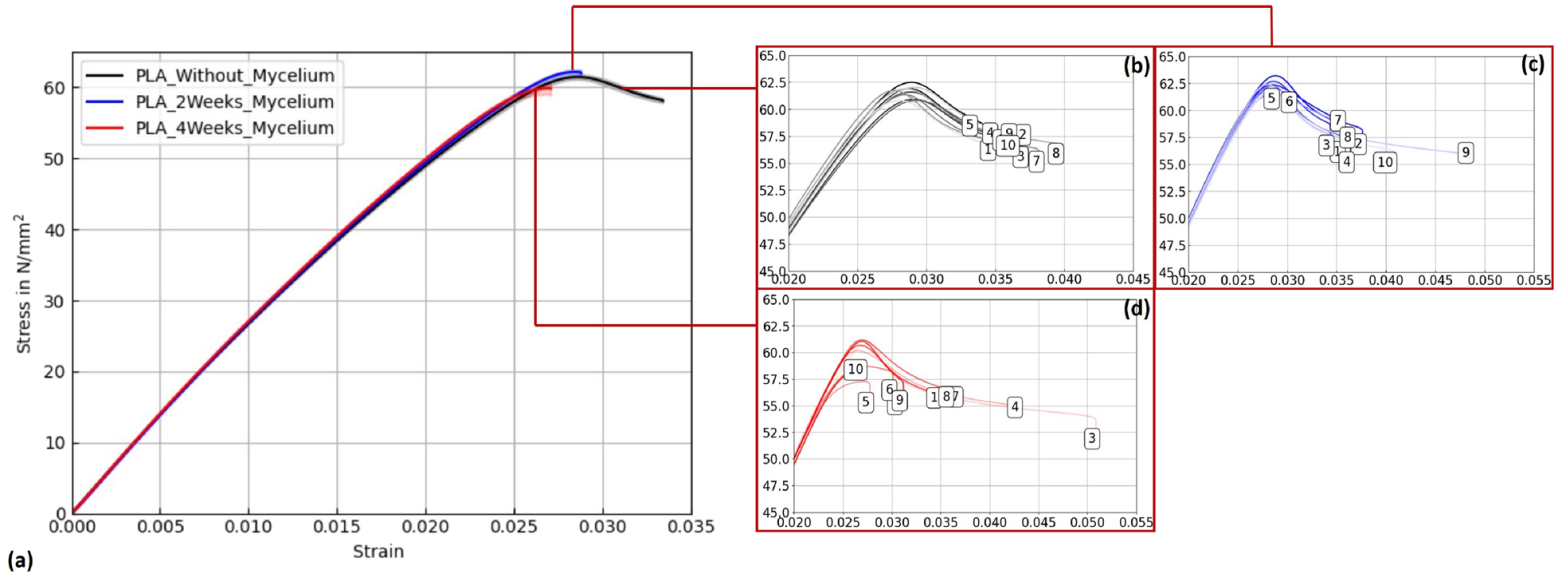
**a** Mean stress-strain diagram for PLA 1 (TruePLA Purple Transparent, Pattern I), showing changes due to mycelium colonization. **b**–**d** Individual curves for untreated, 2-week, and 4-week colonized specimens

**Fig. 17:**
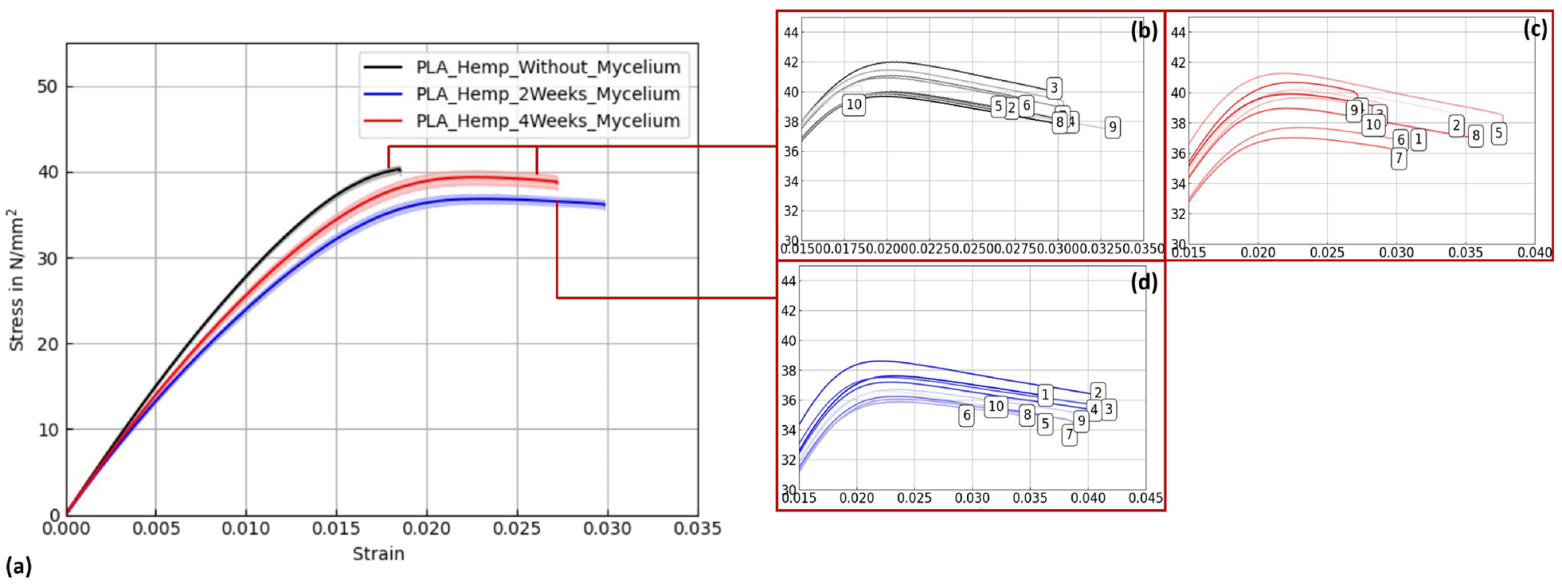
**a** Mean stress-strain diagram for PLA_Hemp specimens (Pattern I). **b**–**d** Individual curves for untreated, 2-week, and 4-week colonized specimens

Figures 15, 16, and 17 display stress–strain curves of specimens 3D-printed with Pattern I, highlighting their mechanical behavior. Two PLA filaments were used: PLA 1 (TruePLA Purple Transparent) and PLA 2 (Prusament PLA Galaxy Silver). Table 7 summarizes the mechanical properties of the specimens, including Young’s modulus and ultimate tensile strength (UTS) with their respective standard deviations (SD), showing the changes observed over different mycelium colonization durations. For PLA 2, uncolonized controls had a mean Young’s modulus of 3015.40 N/mm^2^. After two weeks of colonization, this decreased slightly to 2987.00 N/mm^2^ (–0.9%), and after four weeks to 2975.32 N/mm^2^ (–1.3%). This indicates a minor stiffness reduction with longer colonization.

PLA 1 specimens showed a distinction based on control handling. Controls not fixed at the cultivation box lid, but kept under the same humidity and temperature, exhibited a mean Young’s modulus of 2867.36 N/mm^2^ (see Table ??), whereas specimens fixed at the lid (to avoid mycelium contact) had a slightly lower modulus of 2825.05 N/mm^2^. After two weeks of colonization, the modulus slightly decreased to 2844.24 N/mm^2^ (–0.8%), and after four weeks to 2858.52 N/mm^2^ (–0.3%).

Similarly, PLA_Hemp specimens showed two different control groups: those not fixed at the lid with a mean Young’s modulus of 3072.42 N/mm^2^ (see Table ??), and those fixed at the lid with 3021.36 N/mm^2^. After two weeks of colonization, the modulus significantly dropped to 2694.86 N/mm^2^ (–10.8%). After four weeks, a partial recovery to 2824.25 N/mm^2^ was observed, still 6.5% lower than the fixed control but 4.8% higher than the two-week colonized specimens.

**Table 7:** Mechanical properties (Young’s modulus and ultimate tensile strength, UTS) of PLA 1, PLA 2, and PLA_Hemp specimens with Pattern I: non-colonized, colonized for two weeks, and colonized for approximately four weeks. Values are given as mean ± standard deviation (SD)

Specimen	Young’s modulus		Ultimate tensile strength (UTS)	
	Mean	SD	Mean	SD
	(N/mm^2^)	(N/mm^2^)	(N/mm^2^)	(N/mm^2^)
PLA_Prusament	3015.40	20.60	59.49	0.48
PLA_2W_Prusament	2987.00	11.68	60.20	0.31
PLA_4W_Prusament	2975.32	16.30	60.30	0.18
PLA_TruePLA	2825.05	16.34	61.55	0.18
PLA_2W_TruePLA	2844.24	9.39	62.20	0.17
PLA_4W_TruePLA	2858.52	7.27	59.92	0.43
PLA_Hemp	3021.36	10.61	40.52	0.25
PLA_Hemp_2W	2694.86	22.77	36.82	0.28
PLA_Hemp_4W	2824.25	36.96	39.36	0.44

**Fig. 18:**
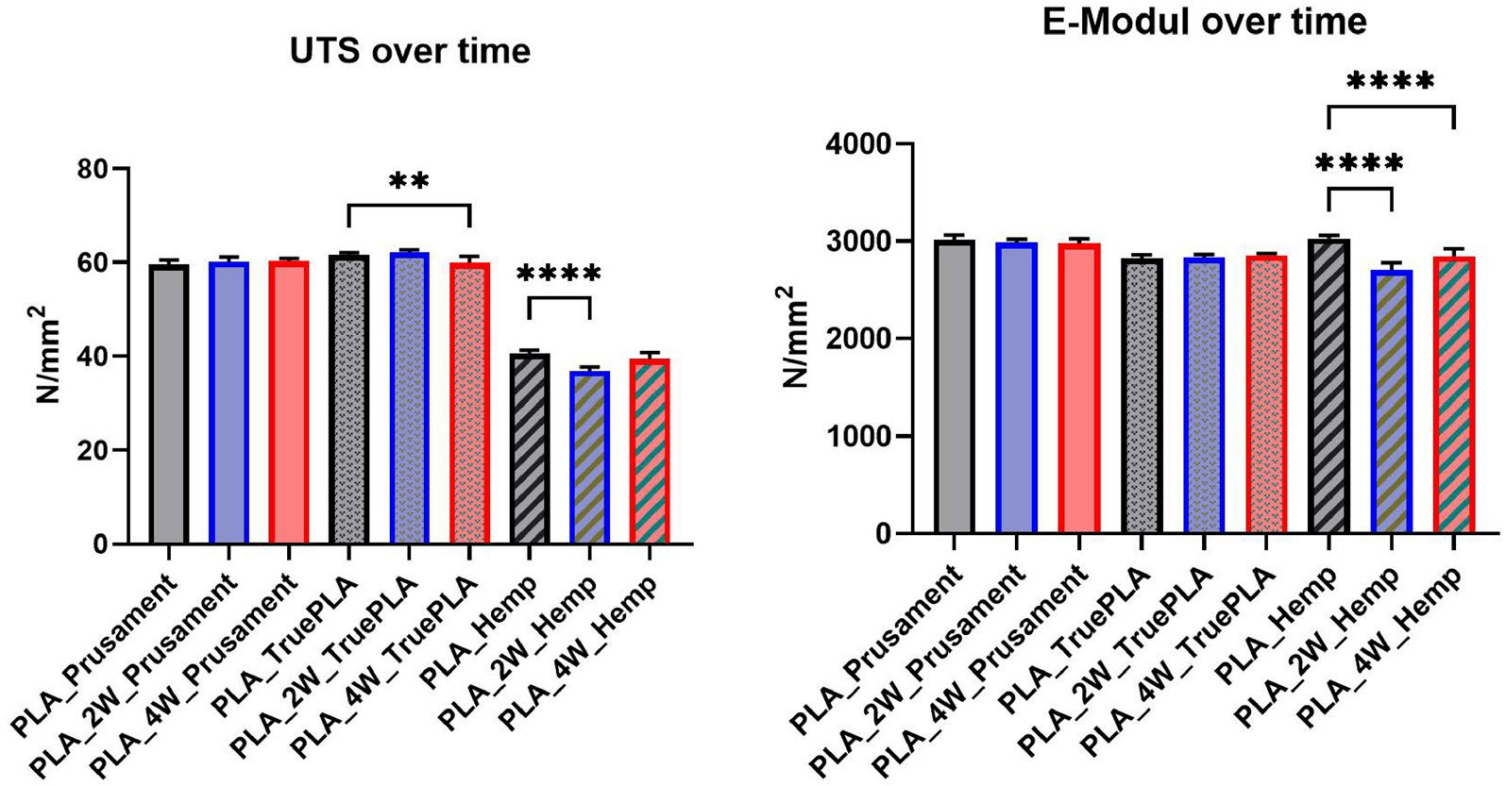
Ultimate tensile strength (left) and mean Young’s modulus (right) of PLA and PLA_Hemp specimens over different colonization times. PLA specimens maintained stable UTS values, whereas PLA_Hemp specimens showed a significant UTS decrease after 2 weeks (****p < 0.0001) followed by partial recovery after 4 weeks. For the E-modulus, significant reductions were observed between PLA_Hemp and both PLA_2W_Hemp and PLA_4W_Hemp (****p < 0.0001), indicating a pronounced effect of colonization on stiffness

To verify statistical relevance of these trends, a one-way ANOVA with Tukey’s test was performed. Results are summarized in Fig. 18. PLA specimens maintained stable UTS values, whereas PLA_Hemp specimens showed a significant UTS decrease after 2 weeks (****p < 0.0001) followed by partial recovery after 4 weeks. No significant changes (ns) were observed in stiffness.

### Microscopic analysis

**Fig. 19:**
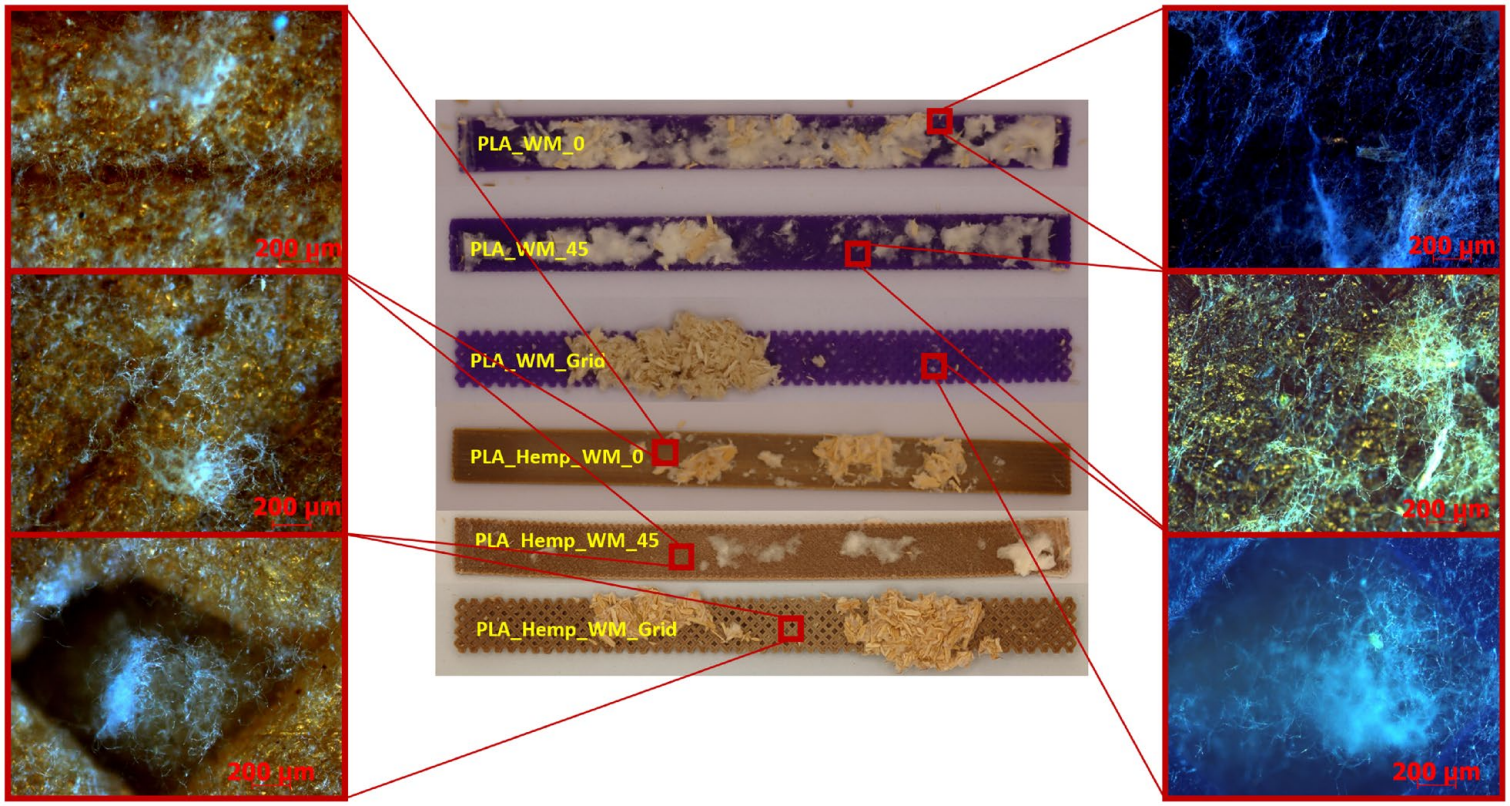
Macroscopic top (center) and corresponding microscopic surface images (left and right) of 3D-printed PLA and PLA_Hemp specimens with Pattern I, II, and III after mycelium colonization. The microscopic images show differences in mycelial surface growth that correlate with the infill pattern geometry

Microscopic images of PLA and PLA_Hemp specimens, shown in Fig. 19 and captured using an Olympus EX51 microscope equipped with an AxioCam MRc camera (Carl Zeiss, Germany) at 50$\times$ magnification, provide insight into the surface-level interaction between mycelium and the printed materials. Mycelial growth was observed primarily on the outer surfaces of the specimens. In one case, fungal hyphae were also found extending into rhombic voids formed by the infill geometry, particularly in Pattern III structures. However, there was no clear evidence of mycelium penetrating between individual PLA deposition layers. Variations in surface coverage appear to be influenced by the geometry and surface roughness of the printed pattern rather than by material composition alone.

## Discussion

The mechanical behavior of PLA and PLA_Hemp specimens exposed to mycelium colonization reveals a complex interplay between material composition, exposure time, and fungal activity. This study aimed to assess whether fungal colonization significantly compromises the structural integrity of 3D-printed biopolymer parts, particularly in the context of future hybrid biocomposite applications. Fungal mycelium has been shown to influence the mechanical properties of polymeric or lignocellulosic substrates through enzymatic activity and moisture uptake [2], while also offering potential as a source for bio-based composite materials [1].

PLA specimens showed varied behavior depending on filament type and internal geometry. Some configurations even exhibited slight stiffness gains, such as an increase of 2.9% in Pattern I and 14.4% in grid geometry, while others, such as Prusament PLA Galaxy Silver, experienced a modest reduction in Young’s modulus. These differences are likely driven by chemical additives, pigmentation, or surface morphology inherent to each filament type, which in turn influence fungal attachment, moisture interaction, and potential interfacial bonding. Comparable trends have been reported by [47, 48], showing that infill geometry and density significantly influence the mechanical performance of MEX AM-printed PLA specimens. Importantly, in the present study none of the PLA variants exhibited major mechanical degradation, underscoring their fundamental resilience to fungal exposure.

In contrast, PLA_Hemp specimens consistently showed a decrease in stiffness following colonization. The strongest decline occurred in grid specimens (–4.7%), with smaller losses in Pattern I and Pattern II variants. A marked reduction after two weeks (–10.8%) followed by partial recovery (–6.5% at four weeks) suggests time-dependent effects. The porous structure introduced by hemp fibers may facilitate fungal infiltration and local degradation, weakening the fiber–matrix interface. At the same time, prolonged exposure may promote denser mycelial coverage, possibly filling voids or contributing structurally in a limited way. Similar observations have been made in natural fiber composites exposed to fungal growth, where reinforcement-matrix interactions are affected by biological activity [1].

Microscopic observations (see Fig. 19) further support these findings and provide morphological evidence for the mechanical trends observed. In PLA specimens, the mycelial growth remained mostly superficial, without clear penetration between the printed layers, which is consistent with their minimal mechanical changes. By contrast, PLA_Hemp samples exhibited rougher surfaces, microvoids, and visible fiber–matrix gaps where fungal hyphae attached and partially infiltrated. This morphology explains the greater stiffness reduction and time-dependent recovery observed in PLA_Hemp, as localized degradation and subsequent mycelial filling affected the interfacial regions. Furthermore, Pattern III specimens with grid geometry showed hyphal extension into rhombic voids, indicating that infill architecture influences fungal attachment and may contribute to anisotropic mechanical behavior. These morphology–mechanical correlations underline the importance of interfacial design and surface topography for future biohybrid composite optimization.

To separate biological effects from purely environmental ones, non-colonized control specimens were incubated under identical conditions. Slight but consistent differences, especially between lid-fixed and unfixed variants, confirm that passive exposure (e.g., moisture absorption, polymer relaxation) can affect mechanical performance. These effects were typically of similar magnitude to those observed in colonized specimens. A notable exception is the autoclaved condition, which caused pronounced mechanical weakening and should be avoided in future workflows [49].

These findings are significant: contrary to common assumptions about biodegradation risks, PLA filaments largely retain their mechanical function even after sustained fungal exposure. This opens the door for the development of hybrid biocomposite structures in design and architecture. The results provide experimental justification for such systems, in which the mechanical role of the base polymer remains intact while the fungal organism contributes additional functional, aesthetic, or regenerative properties.

Earlier work has demonstrated that 3D-printed gyroid scaffolds composed of wood-PLA can be colonized by mycelium, resulting in composites that combine thermal insulation with improved mechanical performance [8]. In these studies, the scaffolds were placed in malt extract agar (MEA), colonized by *Ganoderma lucidum*, and subsequently evaluated with respect to their mechanical and thermal properties. Such findings emphasize the potential of mycelium-based composites for structural applications. By contrast, our results show only minor effects in pure PLA, while PLA_Hemp displays more pronounced, time-dependent variations. This divergence points to an important research gap: the role of scaffold composition (e.g., PLA with different natural fillers) and geometry (e.g., porosity, graded structures) in determining the balance between reinforcement and long-term degradation. Closing this gap in future investigations will be crucial to fully exploit the potential of mycelium-based hybrid composites.

In summary, the mechanical impact of mycelium colonization is modest and often comparable to environmental effects. Natural fibers such as hemp increase the complexity of the interaction, especially in terms of moisture dynamics and biodegradability. For successful material integration, fungal compatibility and environmental sensitivity must be considered at the design stage. PLA-based mycelium composites show promise, but optimization requires a material-specific and context-aware approach. Incorporating these insights alongside literature findings strengthens the understanding of biohybrid composite behavior under fungal colonization.

## Conclusion

This research investigated the influence of mycelial colonization on the mechanical properties of 3D-printed PLA and PLA_Hemp specimens with different infill patterns. The primary objective was to determine whether fungal growth compromises structural integrity, particularly in the context of developing hybrid biocomposite systems.

- The overall mechanical impact of mycelial colonization on PLA was insignificant. Some variants even showed slight improvements in stiffness, with Young’s modulus increasing by up to 2.9% in Pattern I and 14.4% in grid geometry. Ultimate tensile strength remained largely stable, e.g., Prusament PLA increased from 59.49 N/mm^2^ to 60.30 N/mm^2^ after four weeks.
- PLA_Hemp was more susceptible: the incorporation of natural fibers led to moderate reductions in stiffness, particularly in grid specimens (–4.7%) after colonization. Ultimate tensile strength decreased from 40.52 N/mm^2^ to 36.82 N/mm^2^ after two weeks, with partial recovery to 39.36 N/mm^2^ at four weeks, indicating dynamic, time-dependent interactions.
- Environmental exposure, such as humidity and incubation time, also affected mechanical behavior, sometimes to a degree comparable to fungal activity. However, autoclaving caused significant weakening and should be avoided as a pretreatment.
- Overall, PLA filaments maintained structural integrity despite fungal colonization, providing essential evidence for the development of functional biohybrid materials.

These findings confirm the feasibility of constructing more complex, composite structures: the core framework can remain mechanically stable while the mycelium contributes functional, regenerative, or aesthetic properties. By including quantitative results, this conclusion highlights the key trends and provides clearer guidance for future design and material selection.

It has been shown that 3D-printed gyroid scaffolds made of wood-PLA can exhibit substantial reinforcement through mycelial colonization, with yield strength improvements of up to 78% [8]. In contrast, our results indicate only insignificant effects in pure PLA, while PLA_Hemp shows more dynamic, time-dependent changes. This discrepancy highlights a critical research gap concerning the role of bio-based fillers and overall scaffold composition in enabling effective mycelium–polymer integration. Future studies should therefore systematically investigate how different natural fiber reinforcements and biopolymer blends influence colonization dynamics and the resulting mechanical performance.

Ultimately, this work highlights both opportunities and challenges, paving the way for sustainable materials design and construction.

## Data Availability

Data for the additive manufacturing specimens presented in this work are available on GitHub: https://github.com/SVFS-TUBerlin/Mechanical-characterization-of-biopolymer-reinforced-composites . Raw experimental data can be provided upon request from the corresponding author.

## Materials availability

All materials used in the study are commercially available or described within the manuscript. No new materials were developed for this study.
